# Dietary arachidonic acid increases deleterious effects of amyloid-β oligomers on learning abilities and expression of AMPA receptors: putative role of the ACSL4-cPLA_2_ balance

**DOI:** 10.1186/s13195-017-0295-1

**Published:** 2017-08-29

**Authors:** Mélanie H. Thomas, Cédric Paris, Mylène Magnien, Julie Colin, Sandra Pelleïeux, Florence Coste, Marie-Christine Escanyé, Thierry Pillot, Jean-Luc Olivier

**Affiliations:** 1 Research unit on Animals and Functionality of Animal Products (URAFPA), Lorraine University, EA 3998, USC INRA 0340, 2, Avenue de la Forêt de Haye, TSA40602, F-54518 Vandœuvre-lès-Nancy, France; 2 Laboratory of Biomolecules Engineering (LIBio), Lorraine University, 2, Avenue de la Forêt de Haye, TSA40602, F-54518 Vandœuvre-lès-Nancy, France; 3 Biochemistry Department, Central Hospital, University Hospitals of Nancy, 24, avenue du Mal de Lattre de Tassigny, CO n°34, F-54018 Nancy, France; 4 Synaging SAS, 2, rue du Doyen Marcel Roubault, 54518 Vandoeuvre-les-Nancy, France

**Keywords:** Alzheimer’s disease, Amyloid-β oligomers, Arachidonic acid, Diet, Phospholipid species, AMPA receptors, Acyl-CoA synthetase 4, Cytosolic phospholipase A_2_

## Abstract

**Background:**

Polyunsaturated fatty acids play a crucial role in neuronal function, and the modification of these compounds in the brain could have an impact on neurodegenerative diseases such as Alzheimer’s disease. Despite the fact that arachidonic acid is the second foremost polyunsaturated fatty acid besides docosahexaenoic acid, its role and the regulation of its transfer and mobilization in the brain are poorly known.

**Methods:**

Two groups of 39 adult male BALB/c mice were fed with an arachidonic acid-enriched diet or an oleic acid-enriched diet, respectively, for 12 weeks. After 10 weeks on the diet, mice received intracerebroventricular injections of either NaCl solution or amyloid-β peptide (Aβ) oligomers. Y-maze and Morris water maze tests were used to evaluate short- and long-term memory. At 12 weeks on the diet, mice were killed, and blood, liver, and brain samples were collected for lipid and protein analyses.

**Results:**

We found that the administration of an arachidonic acid-enriched diet for 12 weeks induced short-term memory impairment and increased deleterious effects of Aβ oligomers on learning abilities. These cognitive alterations were associated with modifications of expression of α-amino-3-hydroxy-5-methyl-4-isoxazolepropionic acid receptors, postsynaptic density protein 95, and glial fibrillary acidic protein in mouse cortex or hippocampus by the arachidonic acid-enriched diet and Aβ oligomer administration. This diet also led to an imbalance between the main ω-6 fatty acids and the ω-3 fatty acids in favor of the first one in erythrocytes and the liver as well as in the hippocampal and cortical brain structures. In the cortex, the dietary arachidonic acid also induced an increase of arachidonic acid-containing phospholipid species in phosphatidylserine class, whereas intracerebroventricular injections modified several arachidonic acid- and docosahexaenoic acid-containing species in the four phospholipid classes. Finally, we observed that dietary arachidonic acid decreased the expression of the neuronal form of acyl-coenzyme A synthetase 4 in the hippocampus and increased the cytosolic phospholipase A_2_ activation level in the cortices of the mice.

**Conclusions:**

Dietary arachidonic acid could amplify Aβ oligomer neurotoxicity. Its consumption could constitute a risk factor for Alzheimer’s disease in humans and should be taken into account in future preventive strategies. Its deleterious effect on cognitive capacity could be linked to the balance between arachidonic acid-mobilizing enzymes.

**Electronic supplementary material:**

The online version of this article (doi:10.1186/s13195-017-0295-1) contains supplementary material, which is available to authorized users.

## Background

Alzheimer’s disease (AD) and other senile dementias represent a major health problem worldwide, not only in high-income but also in low-income countries, because the number of elderly people is increasing. Despite tremendous work and clinical trials, there is currently no efficient therapy against these diseases. The many failures encountered in the fight against these diseases may be due to the facts that they operate silently over a period of 10 years or more and that therapies are applied after the occurrence of clinical signs, when brain injuries are very advanced. In this context, and considering the high economic and social costs of these diseases, the development of preventive strategies appears to be necessary to delay the occurrence of these diseases. Better knowledge of risk or protective factors is a first step in the design of these preventive strategies. Food components such as fatty acids represent the first modifiable factor. Whereas the role of ω-3 fatty acids and particularly that of the longest one, docosahexaenoic acid (DHA), have been the subject of many studies in the context of AD, the impact of dietary arachidonic acid (ARA) and other ω-6 fatty acids is considerably less well known.

ARA is the second most prevalent polyunsaturated fatty acid (PUFA) in the brain membrane phospholipids besides the neuroprotective DHA. Once released from these phospholipids, free ARA is involved in synaptic function at several levels. It modulates exocytosis of the presynaptic neurotransmitters by inducing soluble *N*-ethylmaleimide-sensitive factor attachment protein receptor formation through its interactions with syntaxin-1A and Munc18 [1, 2]. Studies in zebrafish evidenced that ARA also controls neuronal branch growth and synapse formation by acting as a retrograde messenger [3, 4]. In rat hippocampus, ARA released from the postsynaptic terminal facilitates the presynaptic exocytosis of glutamate [5] and acetylcholine [6] neurotransmitters. More recently, Carta et al. [7] showed that ARA is released in an activity-dependent manner from postsynaptic hippocampal CA3 pyramidal cells and tunes synaptic transmission by modulating presynaptic voltage-gated potassium channels. In addition to its involvement in synaptic plasticity and transmission, free ARA plays a crucial role in neuroinflammation through its conversion into various eicosanoids by cyclooxygenases, prostaglandin synthases, and lipoxygenases, whose activities have been associated with neurodegenerative diseases [8, 9]. In the majority of the studies, researchers have assumed that the production of eicosanoids would contribute to synaptic loss in AD [10], although some positive effects have also been described [11].

ARA is specifically released from membrane phospholipids by cytosolic phospholipase A_2_ (cPLA_2_), which is translocated to the membranes in a cytosolic calcium-dependent manner after its phosphorylation on Ser505 by mitogen-activated protein kinases [12]. We and others [13–15] have shown that cPLA_2_ is activated by amyloid-β peptide (Aβ) oligomers and that its pharmacological inhibition or the suppression of its expression protects neuronal cells against the neurotoxicity of Aβ oligomers and preserves cognitive abilities. According to the Lands cycle [16], ARA released by cPLA_2_ can be reincorporated into the membranes by lysophospholipid acyl transferases after its activation into arachidonoyl coenzyme A (CoA) by acyl-CoA synthetases. Among these enzymes, acyl coenzyme A synthetase 4 (ACSL4) preferentially uses ARA as a substrate and is expressed in neuronal cells as a neuron-specific isoform [17]. To our knowledge, it is not known whether ACSL4 expression or activity is modified by Aβ oligomers. Over the last few decades, numerous studies have clearly shown that Aβ oligomers play a pivotal role in AD through early cognitive alterations by targeting hippocampal synapses [18]. The identity of the receptor(s) of these oligomers on neuronal membranes is still a subject of debate, with more than 15 proteins having been proposed for the binding of Aβ oligomers [18]. The concept of formation of a signaling complex between Aβ oligomers and the cellular prion protein (PrPc) has emerged recently. PrPc physically binds Aβ oligomers and interacts with several molecular actors, such as metabotropic glutamate receptor 5 [19] and LRP1 [20]. PrPc also activates cPLA_2_ in association with its localization in membrane rafts, emphasizing the importance of this ARA-releasing enzyme in the response to Aβ oligomers [21]. Besides the metabotropic glutamate receptor 5, Aβ oligomers affect the activity and recycling of the *N*-methyl-d-aspartate (NMDA) and α-amino-3-hydroxy-5-methyl-4-isoxazolepropionic acid (AMPA) receptors [22, 23]. Inhibition of long-term potentiation (LTP) and induction of long-term depression by Aβ oligomers are considered the putative cellular basis of the memory alterations observed in AD and could be mediated by increased internalization and reduced phosphorylation level of the glutamate receptor 1 (GluR1) AMPA receptors [24].

In this study, we investigated the effect of dietary ARA on the sensitivity of mice to Aβ_42_ oligomers. We show that an increase of ARA intake induced cognitive alteration and increased the neurotoxicity of Aβ_42_ oligomers. To explain these effects, we determined how this diet modified (1) fatty acid composition in peripheral and brain tissues, (2) expression levels of synaptic markers and AMPA receptors, and (3) cPLA_2_ and ACSL4 balance.

## Methods

### Animal handling

All experiments were performed in accordance with the European Union (2010/63/EU) and French directives (2013/118) for animal experimentation regarding the use and care of laboratory animals, as well as with the 3 Rs requirements for animal welfare. The experimental protocols were approved by the regional ethics committee of Lorraine (CELMEA project number 02683.02). Animals were housed according to a 12-h/12-h light/dark cycle with a mean temperature of 21–22 °C and relative humidity of 50 ± 20%, and they were provided either of the two different diets and water ad libitum. Male BALB/c mice 6 weeks of age were provided by Janvier Labs (Le Genest St-Isle, France) and housed in the animal facilities of our laboratory for a 2-week adaptation period. The mice were fed the Harlan Laboratories 2018 standard diet (Teklad Diets/Envigo, Madison, WI, USA) during this adaptation period, before the beginning of the experiments. At 8 weeks of age, the mice were fed ad libitum for 12 weeks with one of two diets (*n* = 39 per group; named *ARA* for arachidonic acid and *OLE* for oleic acid, which will be explained in the next section on diet design). After 10 weeks of the diet, Aβ_42_ oligomers (0.5 nmol in 1 μl) or vehicle (0.15 M NaCl) were injected into the brain’s right ventricle of anesthetized mice (NaCl-injected OLE mice, *n* = 19; NaCl-injected ARA mice, *n* = 19; Aβ_42_-injected OLE mice, *n* = 20; Aβ_42_-injected ARA mice, *n* = 20) according to the stereotactic coordinates from the bregma (anteroposterior, −0.22 mm; lateral, −1 mm; dorsal, +2.5 mm). Injections were done using a 10-μl Hamilton microsyringe fitted with a 26-gauge needle. All surgical procedures were performed on anesthetized mice (ketamine-xylazine mix, 1 mg/10 g body weight, intraperitoneal injection). The cognitive performance of the mice was tested 3–14 days after the injections. Blood was collected from the submandibular vein twice: first at the end of the adaptation period, before the initiation of the OLE and ARA diets, and then at the end of the 12 experimental weeks, 2 weeks after Aβ oligomer or control intracerebroventricular (icv) injections and just before the mice were killed. Blood pellets and plasma samples were stored at −80 °C after separation and before biochemical analyses. Twenty-week-old BALB/c mice were killed by isoflurane inhalation (Mundipharma, Paris, France). The liver, cerebral cortex, and hippocampus were collected, rapidly frozen in liquid nitrogen, and stored at −80 °C until biochemical analysis.

### Diet design

Two diets were tested in this study. One of them, the ARA diet, was a ω-6 acid- and ARA-rich diet and was prepared with 20 g/kg hemp oil (Earl de la Fermes d’Ormes, Ormes, France), 55 g/kg lard (Scientific Animal Food & Engineering, Augy, France), and 25 g/kg ARASCO oil (DSM Nutritional Products, Courbevoie, France) added to the 210 control diet (Scientific Animal Food & Engineering). In the other diet, the OLE-rich diet, OLE replaced ARA, whereas the amounts of the other fatty acids remained similar to those in the ARA diet. The OLE diet was prepared with sunflower (5 g/kg) and rape (30 g/kg) oils (Earl de la Fermes d’Ormes) and lard (65 g/kg; Scientific Animal Food & Engineering). Diet composition was calculated by gas chromatography (GC) analysis of the various oils and lard and checked after preparation by Scientific Animal Food & Engineering. The proportions of proteins, carbohydrate, and fat, as well as the fatty acid composition, of each diet are shown in Table 1. The two diets contained 10% fat and similar amounts of saturated stearic and palmitic acids: 33.4% (OLE diet) and 32.9% (ARA diet) with ratios palmitic acid/stearic acid of 2.1 and 1.9, respectively (Table 1). Compared with the standard diet (Teklad Diets), which is commonly used in our laboratory and contains 6.2% lipids, the OLE and ARA diets provided approximatively threefold higher amounts of saturated fatty acids (Table 1). The OLE and ARA diets also contained 5.7% and 5.3%, respectively, of the ω-3 precursor α-linolenic acid, above the 4.4% found in the standard diet (Table 1). Therefore, the ARA and OLE diets provided approximatively twice the α-linolenic acid per gram of food compared with that in the standard diet. In addition, the ARA and OLE diets provided 0.9% and 0.2% DHA, respectively, whereas the standard diet did not contain any ω-3 PUFA (Table 1). By contrast, the OLE and ARA diets provided between 33% and 50% less linoleic acid (ω-6 precursor). Diets were stored at 4 °C and protected from light to prevent oxidation.

**Table 1:** Composition of the standard, oleic acid-enriched, and arachidonic acid-enriched diets

	STD	OLE	ARA
Proteins, % of food	18.6	19.6	19.6
Carbohydrate, % of food	44.2	55.1	55.1
Fat, % of food	6.2	10	10
Fatty acid composition (% of measured FA)			
Palmitic acid (16:0)	13.4	22.8^a^	21.6^a,b^
Stearic acid (18:0)	3.4	10.6^a^	11.3^a^
Palmitoleic acid (16:1 ω-7)	0.1	1.8^a^	1.5^a^
Oleic acid (18:1 ω-9)	18.4	43.7^a^	27.3^a,c^
Linoleic acid (18:2 ω-6)	60.1	15^a^	20.4^a^
Arachidonic acid (20:4 ω-6)	0.2	0.2	11.5^a,c^
Docosatetraenoic acid (22:4 n-6)	0.0	0.1^a^	0.1^a,d^
α-Linolenic acid (18:3 ω-3)	4.4	5.7^a^	5.3^e^
Eicosapentaenoic acid (20:5 ω-3)	0.0	0.0	0.0
Docosahexaenoic acid (22:6 n-3)	0.0	0.2^a^	0.9^a^
Sum of ω-6 PUFA	60.2	15.3^a^	32^a,c^
Sum of ω-3 PUFA	4.4	5.9^a^	6.2^a,b^
ω-6/ω-3 PUFA ratio	13.7	2.6^a^	5.2^a,c^

*Abbreviations: ARA* Arachidonic acid-enriched diet, *FA* Fatty acid, *OLE* Oleic acid-enriched diet, *PUFA* Polyunsaturated fatty acid, *STD* Standard diet

^a^ *p* < 0.001, OLE or ARA compared with STD

^b^ *p* < 0.01, ARA compared with OLE

^c^ *p* < 0.001, ARA compared with OLE

^d^ *p* < 0.05, ARA compared with OLE

^e^ *p* < 0.01, OLE or ARA compared with STD

### Preparation of Aβ_42_ oligomers

Aβ_42_ peptides were obtained from Bachem Distribution Services (Weil am Rhein, Germany). To overcome problems of peptide solubility at high concentrations, fresh peptide stock solutions were prepared at 5 mg/ml in the disaggregating solvent hexafluoro-2-propanol and kept frozen at −80 °C. Soluble Aβ_42_ oligomers were prepared as stock solutions at the concentration of 0.5 mM in sterile 0.1 M PBS (pH 7.4), and aliquots were stored at −20 °C until use as previously described [15, 25]. Aβ_42_ oligomers preparations do not contain fibrils; they contain mainly monomers, dimers, trimers, and tetramers (Additional file 1). Each batch of oligomers was checked for cellular toxicity on neuronal cell primary cultures and for their effect on animal cognitive abilities before their use in the experiments.

### Behavioral analyses

#### Y-maze

Immediate working memory performance was assessed by recording spontaneous alternation behavior in a Y-maze as previously described [25]. The Y-maze task was carried out on day 4 after soluble Aβ_42_ oligomer administration. The maze was made of opaque Plexiglas, and the arms were 40 cm long, 16 cm high, 9 cm wide, and positioned at equal angles. Mice were placed at the end of one arm and allowed to move freely through the maze during a 5-minute session. The series of arm entries were recorded visually, and arm entry was considered to be completed when the hind paws of the mouse were completely placed in the arm. Alternation was defined as successive entries into the three arms on overlapping triplet sets. The percentage alternation was calculated as the ratio of actual (total alternations) to possible (defined as the number of arm entries minus two) alternations, multiplied by 100 [26].

#### Morris water maze

The Morris water maze was performed as previously described [27]. The experimental apparatus consisted of a circular water tank (diameter 80 cm, height 50 cm) containing water at 22 °C and rendered opaque by adding an aqueous acrylic emulsion. A platform (diameter 10 cm) was submerged 1 cm below the water surface and placed at the midpoint of one quadrant. The pool was placed in a test room homogeneously brightened at 100 lx and containing various prominent visual cues on the walls. The swimming paths of the animals were recorded using a video tracking system.

On days 3 and 4 after injection, navigation to a visible platform was performed before place navigation to evaluate visual and motor abilities of the animals. Mice were subjected to four trials per day, with two trials in the morning and two trials in the afternoon and an interval between trials of at least 45 minutes. There was no additional maze cue in the room. The platform position and starting points were randomly distributed over all four quadrants of the pool. Mice that failed to find the platform after 60 seconds were guided to its location.

Next, memory acquisition trials (training) were performed four times daily on 5 consecutive days to reach a steady state of escape latency. The mice were allowed to swim freely for 60 seconds, left alone for an additional 30 seconds on the hidden platform, and then returned to the home cage during the intertrial interval. The intervals between the trials were 45 minutes. The start positions, set at each limit between quadrants, were randomly selected for each animal. In each trial, the time required to escape onto the hidden platform was recorded. Mice failing to find the platform within 60 seconds were placed on the platform for 10 seconds at the end of the trial.

Memory retention tests (probe trials) were performed 3 days after the last training session (14 days after injection). The platform was removed, and each mouse was allowed a free 60-second swim. The latency to cross the quadrant of the previous location of the platform and the time spent in each of the four quadrants were measured by replay using a video recorder.

### Biochemical analyses

#### Immunoblot analysis and enzyme-linked immunosorbent assay

Homogenates were prepared from the cortex and hippocampus. Briefly, brain regions were dissected and homogenized in 25 mM Tris-HCl containing 150 mM NaCl, 1 mM ethylenediaminetetraacetic acid (EDTA), 1% (vol/vol) Nonidet P-40, 1% (wt/vol) sodium deoxycholate, and 0.1% (wt/vol) SDS and protease inhibitors (cOmplete protease inhibitor cocktail; Roche, Basel, Switzerland). After homogenization, tissues were lysed using two cycles of freezing and thawing and finally centrifuged at 4 °C for 10 minutes at 10,000 × *g*. The protein concentration in the supernatant was determined using a Pierce bicinchoninic acid protein assay kit (Thermo Fisher Scientific, Waltham, MA, USA). Synaptosomal membrane-enriched fractions were prepared as previously described and were used to evaluate the expression levels of synaptic proteins [26]. The cortex and hippocampus were dissected and rapidly homogenized in ice-cold 10 mM Tris buffer (pH 7.4) containing 0.32 mM sucrose, 2 mM EDTA, 10 μg/ml sodium orthovanadate, and protease inhibitors (cOmplete protease inhibitor cocktail). The homogenate was spun at 800 × *g* for 15 minutes to remove nuclei and cell debris. The resulting supernatant was then centrifuged at 12,000 × *g* for 20 minutes, and a second pellet was collected.

Samples (10 μg) were mixed with an equal volume of 2× Laemmli buffer and denaturated by heating the mixture for 5 minutes at 95 °C. Samples were then subjected to SDS-PAGE and immunoblotting on polyvinylidene difluoride membranes. The following antibodies were used: AMPA receptors GluR1–GluR4 (1:1000 dilution; Cell Signaling Technology, St-Quentin-en-Yvelines, France), postsynaptic density protein 95 (PSD95) (1:1000 dilution; Cell Signaling Technology), glial fibrillary acidic protein (GFAP) (1:1000 dilution; Merck Millipore, Molsheim, France), TREM-2 (1:500 dilution; Abcam, Paris, France), phosphorylated cytosolic phospholipase A_2_ (P-cPLA_2_) (1:1000 dilution; Cell Signaling Technology), cPLA_2_ (1:1000 dilution; Cell Signaling Technology), ACSL4 (1:1000 dilution; Santa Cruz Biotechnology, Heidelberg, Germany), and β-tubulin (1:10,000 dilution; Sigma-Aldrich Chemie, St-Quentin Fallavier, France). Immunoblots were probed with the corresponding HRP-conjugated secondary antibodies (1:2000 dilution; Cell Signaling Technology) and detected using an enhanced chemiluminescence protocol (SuperSignal HRP Substrate; Merck Millipore). The FUSION FX5 system (Vilber Lourmat, Marne-la-Vallée, France) was used to detect chemiluminescence, and ImageJ software (National Institutes of Health, Bethesda, MD, USA) was used to quantify the protein levels.

Concentrations of the Iba1/AIF-1 protein and the GluR3 and GluR4 AMPA receptors were measured in the cortex and hippocampus homogenates by using specific murine enzyme-linked immunosorbent assay (ELISA) kits from Cloud-Clone Corporation (Katy, TX, USA). Specific kits furnished by Aviva Systems Biology Corporation (San Diego, CA, USA) were used to evaluate concentrations of the murine GluR1 and GluR2 AMPA receptors. All the ELISA measurements were performed according the furnisher’s recommendations.

#### Cholesterol and triglyceride measurements

Blood samples were collected in tubes containing a 5% EDTA solution and were centrifuged at 12,000 × *g* to separate plasma from erythrocytes, which were preserved at −80 °C. Cholesterol and triglyceride concentrations were measured with enzymatic activity identification kits (cholesterol RTU and triglyceride RTU; bioMérieux, Marcy-l’Étoile, France France) in eight plasma samples in each of the four mouse subgroups (NaCl-injected OLE mice, Aβ_42_-injected OLE mice, NaCl-injected ARA mice, and Aβ_42_-injected ARA mice).

#### Fatty acid GC analysis

Lipids were extracted from erythrocytes and liver and brain structures according to the Folch protocol [28]. Erythrocyte lipids were extracted by mixing 100 μl of erythrocyte pellets with 3 ml of chloroform and isopropanol. After mixing, upper and lower phases were separated at 1500 × *g* for 10 minutes at 4 °C. The upper phases were recovered and evaporated under a nitrogen gas stream. Then, the various lipid extracts were prepared by transesterification as described by Lepage and Roy [29]. Transesterification reactions were stopped by potassium carbonate, strongly homogenized, and finally centrifuged at 4 °C for 10 minutes at 2000 × *g*. The supernatants were recovered and were added to 2 ml of acetonitrile. Then, the mixtures were strongly homogenized and centrifuged at 4 °C for 10 minutes at 2000 × *g*. The upper phases were recovered and evaporated under a continual stream of nitrogen gas. Samples were then diluted in 50 μl of hexane and injected into a Clarus 480 gas chromatograph (PerkinElmer, Waltham, MA, USA) provided with an automatic injector. The esterified fatty acids were separated in a DB-23 column [(50%-cyanopropyl-phenyl)-methylpolysiloxane; Agilent Technologies, Massy, France] and detected with flame ionization. The temperature of the injector was fixed at 270 °C, and that of the detector was fixed at 280 °C. The column oven temperature, originally at 170 °C, was gradually increased by 2.8 °C/minute, then by 0.1 °C/minute, to reach 230 °C. The vector gas was nitrogen with a pressure of 400 kPa. The pressure at the top of the column was 40 kPa. The peaks obtained were integrated by using the EZChrom Elite software (Agilent Technologies) and quantified by using a standard range between 25 and 100 μg/ml of the main fatty acids (palmitic, palmitoleic, stearic, oleic, linolenic, linoleic, arachidonic, eicosapentaenoic, docosatetraenoic, and docosahexaenoic acids; Sigma-Aldrich Chemie). The results are presented as percentages of these ten fatty acids.

#### Phospholipid analysis by liquid chromatography-tandem mass spectrometry

After extraction and evaporation of the solvent, lipids were diluted in 200 μl of choloroform:methanol (2:1), and 16 μl of each sample was injected into an ultra-high-pressure liquid chromatography-mass spectrometry system (Thermo Fisher Scientific, San Jose, CA, USA). The lipids were separated on a LiChroCART, LiChrospher 100 DIOL, 250 × 200-mm (5 μm) precolumn (Merck, Darmstadt, Germany). Chloroform was used as mobile phase A. Mobile phase B was methanol with 0.1% (vol/vol) formic acid, and ammonia was added to pH 5.3 (approximately 0.05% vol/vol ammonia) and 0.05% (vol/vol) triethylamine. Separation was accomplished by using a gradient starting at 95% mobile phase A, decreasing to 70% A in 11 minutes, further decreasing to 20% A in 3 minutes, and held at 20% A in 4 minutes and then back to 95% A in 2 minutes. The total chromatographic run time was 30 minutes. The flow rate was 0.3 ml/minute, and the column temperature was 30 °C [30].

After being separated, the compounds of interest were driven by the mobile phase to the source block of the mass spectrometer, where they were ionized in negative electrospray mode. The ionization parameters were as follows. The potential of the electrospray needle was 4.5 kV, and the temperature of the transfer capillary was 230 °C. The ionic optic parameters were optimized by infusion of a standard solution of a phospholipid mixture to maximize the mass spectrometric signal of the compounds of interest. The mass resolution was 0.2 Da. In addition to a mass spectrometric scan between 100 and 2000 mass-to-charge ratio to observe the parent ions, MS2 and MS3 fragmentation was manually performed to obtain structural information on phospholipid classes [30]. The interpretation of the MS2 and MS3 spectra allowed us to know the nature of the species (the fatty acid composition) of each phospholipid.

### Statistical analysis

StatView software was used for the statistical analysis. Multiple pairwise comparisons among the groups of data were performed using analysis of variance followed by Scheffé’s post hoc test. *p* < 0.05 indicated significant differences between experimental groups. All reported values represent the mean ± SEM.

## Results

### Body mass, cholesterolemia/triglyceridemia, and erythrocyte/liver fatty acid composition: variation induced by ARA and OLE diets

**Fig. 1:**
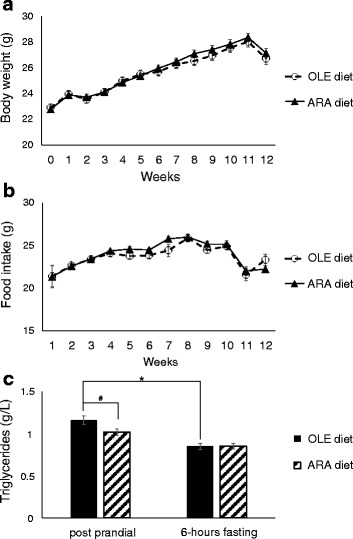
Body weight, food intake, and plasma triglyceride evolution after mice were fed the arachidonic acid-enriched (ARA) diet. Body weight (**a**) and food intake **b** were measured once per week, and data are expressed in grams of body weight of the mice (**a**) and grams of food for their food intake (**b**). Postprandial and 6-h fasting plasma triglycerides **c** were measured after 12 weeks of the ARA or oleic acid-enriched (OLE) diet, and data are expressed in grams per liter (*n* = 16 per group; * *p* < 0.05 comparing the OLE and ARA mice; ^#^ *p* < 0.05 comparing postprandial and 6-h fasting data). All data are presented as mean ± standard error of the mean (SEM)

As detailed in the Methods section above, two groups of thirty-nine 8-week-old male BALB/c mice were fed one of two different diets after a 2-week adaptation period during which they were fed a standard diet. The OLE group was fed a diet rich in OLE as a control diet, and the ARA group was fed an ARA-enriched diet. The body mass gains of the two groups of mice (OLE and ARA) were identical, starting from 22.8 ± 0.2 g at the beginning of the experiments to 28.4 ± 0.3 g at the 11th week (Fig. 1a).During the last week of the experiment (12th week), the mice lost 1.4 ± 0.3 g in relation to a decrease in food intake during the 11th week (Fig. 1b), regardless of the nature of the diets and the injected products (saline solution or Aβ_42_ oligomers). Postprandial and 6-h fasting cholesterolemia and triglyceridemia were measured at the end of the 12 weeks of the diets in plasma of 8 mice per group (16 OLE and 16 ARA mice). Postprandial cholesterolemia was similar in mice fed the OLE or ARA diet (1.76 g/L, on average; *see* Additional file 2). Fasting for 6 h decreased cholesterolemia by 11% in the same way in both diet groups (*see* Additional file 2). In contrast, postprandial triglyceridemia decreased by 11–13% in mice fed the ARA diet compared with those fed the OLE diet (Fig. 1c). Fasting triglyceridemia was identical in both diet groups (Fig. 1c).

**Fig. 2:**
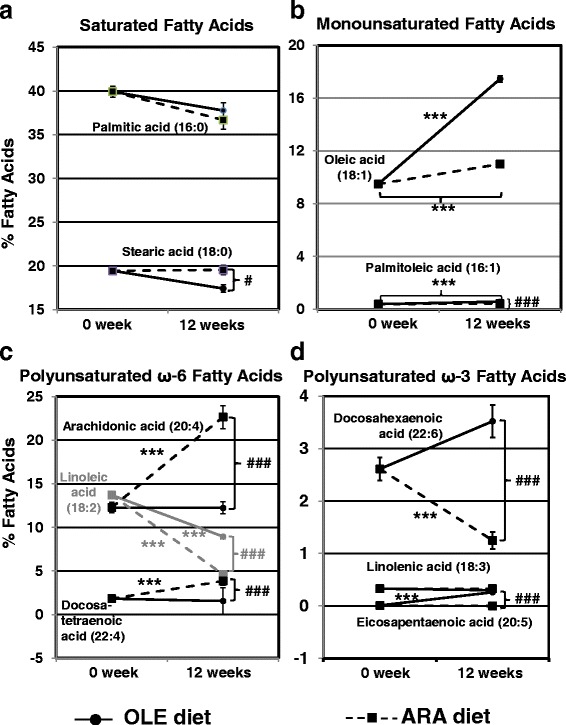
Modifications of erythrocyte fatty acid composition by oleic acid-enriched (OLE) and arachidonic acid-enriched (ARA) diets. Mice were fed a standard diet for 2 weeks before the experiments. Blood samples were collected, and then two groups were fed the OLE or ARA diet for 12 weeks. Second blood samples were collected at the end of the 12th week. Fatty acid composition was measured by gas chromatography to evaluate the evolution of the fatty acid profiles between the beginning and the end of the experiments in the OLE- or ARA-fed mice. Saturated fatty acids (**a**), monounsaturated fatty acids (**b**), polyunsaturated ω-6 fatty acids (**c**), and polyunsaturated ω-3 fatty acids (**d**) were measured. * *p* < 0.05, ** *p* < 0.01, *** *p* < 0.001, comparison of measures at weeks 0 and 12 in a diet group (OLE or ARA). ^#^ *p* < 0.05, ^##^ *p* < 0.01, ^###^ *p* < 0.001, comparison between OLE- and ARA-fed mice at week 12. *n* = 10 in each diet group (ARA or OLE)

Because the two diets contained more lipids as well as different fatty acid compositions than the standard diet, which was given to our 8-week-old mice for 2 weeks before the beginning of experiments, we examined the variations in fatty acid composition in circulating erythrocytes before and after the experiments (Fig. 2). In erythrocytes, fatty acids are esterified almost only in membrane phospholipids, and their composition faithfully reflects food intake [31]. Regarding the saturated fatty acids, both the OLE and ARA diets slightly decreased the palmitic acid amounts after 12 weeks, whereas only the OLE diet reduced stearic acid amounts, leading to a 2.13% significant difference between the OLE- and ARA-fed mice after 12 weeks on the diet (Fig. 2a). Regarding monounsaturated fatty acids, the oleic acid amount increased from 9.48 ± 0.08% to 11.01 ± 0.16% with the ARA diet, whereas it rose to 17.45 ± 0.24% with the OLE diet (Fig. 2b). The OLE diet also increased the monounsatured palmitoleic acid amount from 0.39 ± 0.02% to 0.58 ± 0.02% (1.5 times), whereas the ARA diet did not significantly modify this fatty acid in erythrocytes compared with the standard diet (Fig. 2b and Table 1). Regarding the ω-6 PUFAs, the ARA diet drastically enhanced the amounts of ARA (from 12.29 ± 0.58% to 22.66 ± 1.32%) and docosatetraenoic acid (DTA) (from 1.83 ± 0.08% to 3.84 ± 0.45%), another ω-6 PUFA (C22:4, ω-6), whereas the OLE diet maintained these fatty acids at the levels found before the experiments (Fig. 2c). By contrast, the amounts of the ω-6 precursor, linoleic acid, were lowered by both the ARA and OLE diets in erythrocytes after 12 weeks of the diet, with the influence of the ARA diet being stronger (from 13.71 ± 0.31% to 8.95 ± 0.24% and 4.61 ± 0.13% with the OLE and ARA diets, respectively). Finally, regarding the ω-3 PUFAs, the OLE diet increased eicosapentaenoic acid (EPA) and DHA amounts in erythrocytes (from 0.01 ± 0.008% to 0.26 ± 0.03% and from 2.61 ± 0.22% to 3.52 ± 0.31%, respectively), whereas the ARA diet decreased DHA amounts to 1.25 ± 0.16% (i.e., half the values observed before the experiments) (Fig. 2d). In the main 2 groups of 39 mice fed the OLE and ARA diets, 2 subgroups of mice were given icv injections of 500 pmol Aβ_42_ oligomers or vehicle (*n* = 20 and 19, respectively) at the end of the 10th diet week, as previously described [15]. No difference was observed at the end of the 12 weeks of the diet between the two subgroups of mice given icv injections of NaCl or Aβ oligomers in each diet group for the erythrocyte fatty acid composition (*see* Additional file 3).

We also investigated the differences in fatty acid composition in the liver, another type of peripheral tissue, in both mice groups (Table 2). In the liver, esterified fatty acids are distributed mainly in triglycerides and cholesterol esters, and only a minor part is found in membrane phospholipids. Liver amounts of stearic acid were higher (1.2 times), whereas palmitic acid amounts were lower (0.93 times) in ARA-fed mice than in OLE-fed mice, which are similar to the differences observed in erythrocytes after 12 weeks of the diet, except that the variations of palmitic acid were not significantly different between the two diets in erythrocytes. Oleic acid amounts were 1.6-fold higher in the livers of OLE-fed mice than in those of ARA-fed mice (Table 2), as in erythrocytes after 12 weeks of the diet (Fig. 2b). Palmitoleic acid, the other monounsaturated fatty acid, was decreased by the ARA diet compared with the OLE diet in liver in the same proportions as in erythrocytes (0.75 times). Regarding ω-6 PUFAs, linoleic acid was only weakly reduced by 15% in the livers of mice fed the ARA diet compared with those fed the OLE diet, which contrasts with the 50% reduction observed in erythrocytes. Liver ARA amounts were enhanced by 1.7 times with the ARA diet compared with the OLE diet, similar to the erythrocyte profiles. By contrast, the increase in DTA amounts with the ARA diet was higher in the liver than in erythrocytes (4.1 instead of 2.5 times) (Table 2 and Fig. 2c). In parallel to the increase of the ω-6 PUFAs, ω-3 PUFA amounts were decreased by the ARA diet compared with the OLE diet in the liver (0.12 and 0.6 times for EPA and DHA, respectively) (Table 2), although the DHA reduction was lower in the liver than in erythrocytes (0.36 times) (Fig. 2d). As in erythrocytes, no difference was observed at the end of the 12 weeks of the diet between the mice given icv injections with NaCl and those given icv injections with Aβ oligomers in each diet group for the liver fatty acid composition (*see* Additional file 4).

**Table 2:** Liver fatty acid composition in mice fed oleic acid-enriched or arachidonic acid-enriched diet (%)

	OLE	ARA
Palmitic acid (16:0)	26.49 ± 0.56	24.55 ± 0.36^a^
Stearic acid (18:0)	13.48 ± 0.39	16.73 ± 0.74^b^
Palmitoleic acid (16:1)	1.25 ± 0.08	0.94 ± 0.06^a^
Oleic acid (18:1)	16.08 ± 1.53	10.09 ± 0.56^b^
Linoleic acid (18:2 ω-6)	15.38 ± 0,87	12.86 ± 0.81^c^
Arachidonic acid (20:4 ω-6)	15.97 ± 0.61	27.46 ± 0.77^b^
Docosatetraenoic acid (22:4 ω-6)	0.47 ± 0.01	1.93 ± 0.16^b^
α-Linolenic acid (18:3 ω-3)	0.41 ± 0.04	0.52 ± 0.05
Eicosapentaenoic acid (20:5 ω-3)	0.48 ± 0.06	0.06 ± 0.01^b^
Docosahexaenoic acid (22:6 ω-3)	10.00 ± 0.27	6.10 ± 0.26^b^
Sum of ω-6 PUFA	31.8	42.25
Sum of ω-3 PUFA	10.89	6.69

*ARA* Arachidonic acid-enriched diet, *OLE* Oleic acid-enriched diet, *PUFA* Polyunsaturated fatty acid

^a^ *p* < 0.01, ARA compared with OLE. *n* = 10 in each diet group (OLE or ARA)

^b^ *p* < 0.001, ARA compared with OLE. *n* = 10 in each diet group (OLE or ARA)

^c^ *p* < 0.05, ARA compared with OLE. *n* = 10 in each diet group (OLE or ARA)

### Modifications of fatty acid composition of brain hippocampus and cortex by dietary ARA

The results reported above show that the ARA and OLE diets lead to large differences in fatty composition in erythrocytes and the liver. Therefore, we investigated whether the fatty acid composition of brain structures, hippocampus, and cortex were affected in the same way by these diets. Compared with the variations observed in erythrocytes and the liver, differences in brain fatty acid composition had more limited amplitudes and were restricted to the OLE diet, the ω-6 fatty acids (linoleic ARA and DTA), and ω-3 PUFA DHA. The ARA diet reduced by 0.95 and 0.92 times the OLE amounts in the cortex and hippocampus, respectively, compared with the OLE diet (Table 3). The amounts of the ω-6 PUFAs ARA, and DTA were higher in the hippocampus (1.12 times for ARA and 1.22 times for DTA) and the cortex (1.1 times for ARA and 1.26–1.29 times for DTA) of mice fed the ARA diet compared with mice fed the OLE diet. The ARA diet also induced a reduction of DHA (0.91 times) in the cortex. DHA amounts trended lower in the hippocampus of ARA-fed mice (0.95 times), but the difference did not reach a statistically significant level. Because brain fatty acid profiles could be changed by the icv Aβ_42_ oligomer injections, we separately examined the four mouse subgroups (OLE-fed mice given saline solution or Aβ_42_ oligomer injections and ARA-fed mice given saline solution or Aβ_42_ oligomer injections). No difference was observed between mice given icv NaCl and Aβ_42_ oligomer injections in each diet group (*see* Additional file 5).

**Table 3:** Cortex and hippocampus fatty acid composition (%) in mice fed oleic acid-enriched or arachidonic acid-enriched diet

	Cortex		Hippocampus	
	OLE	ARA	OLE	ARA
Palmitic acid (16:0)	26.92 ± 0.07	27.04 ± 0.11	25.23 ± 0.14	24.76 ± 0.23
Stearic acid (18:0)	24.93 ± 0.19	25.17 ± 0.09	26.03 ± 0.18	25.72 ± 0.37
Palmitoleic acid (16:1)	0.40 ± 0.00	0.39 ± 0.01	0.43 ± 0.01	0.41 ± 0.00
Oleic acid (18:1)	12.13 ± 0.06	11.52 ± 0.06^a^	12.68 ± 0.16	11.61 ± 0.13^a^
Linoleic acid (18:2 ω-6)	0.61 ± 0.01	0.38 ± 0.02^b^	0.61 ± 0.02	0.34 ± 0.02^a^
Arachidonic acid (20:4 ω-6)	11.50 ± 0.03	12.69 ± 0.05^b^	13.08 ± 0.15	14.65 ± 0.23^a^
Docosatetraenoic acid (22:4 ω-6)	3.71 ± 0.02	4.75 ± 0.04^b^	4.76 ± 0.15	5.82 ± 0.09^b^
Linolenic acid (18:3 ω-3)	LOQ	LOQ	0.05 ± 0.05	0.02 ± 0.02
Eicosapentaenoic acid (20:5 ω-3)	LOQ	LOQ	LOQ	LOQ
Docosahexaenoic acid (22:6 ω-3)	19.81 ± 0.06	18.04 ± 0.10^b^	17.14 ± 0.26	16.68 ± 0.38
Sum of ω-6 PUFA	15.82	17.82	18.44	20.81
Sum of ω-3 PUFA	19.81	18.07	17.19	16.69

*Abbreviations: ARA* Arachidonic acid-enriched diet, *OLE* Oleic acid-enriched diet, *LOQ* Limit of quantitation *PUFA* Polyunsaturated fatty acid

^a^ *p* < 0.05, ARA compared with OLE. *n* = 8 in OLE diet group, *n* = 12 in ARA diet group

^b^ *p* < 0.001, ARA compared with OLE. *n* = 8 in OLE diet group, *n* = 12 in ARA diet group

### Dietary ARA affects cognitive abilities of wild-type BALB/c mice and enhances their alteration by Aβ_42_ oligomers

Because we found evidence of slight variations in fatty acid composition of the hippocampus and cortex of mice fed the ARA diet compared with those fed the OLE diet, we examined whether these differences could be associated with modifications of the basal cognitive abilities and their responses to Aβ_42_ oligomers. As described in the Methods section above, the cognitive performance of the four subgroups of mice was monitored using the Y-maze test, reflecting the immediate spatial working memory performance, and the Morris water maze test, reflecting learning and long-term memory capacities.

**Fig. 3:**
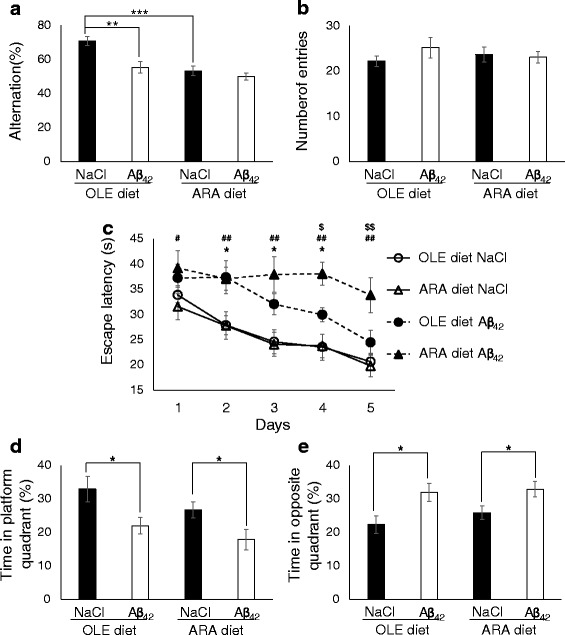
Increased susceptibility of the mice to amyloid-β peptide (Aβ)-induced learning and short-term memory impairment by arachidonic acid-enriched (ARA) diet. Mice were given intracerebroventricular injections of 0.9% NaCl or soluble Aβ_42_ oligomers as described in the Methods section of the main text after 10 weeks of the ARA or oleic acid-enriched (OLE) diet. Y-maze tests were performed 4 days after the injection, and **a** spontaneous alternation behavior and **b** number of arm entries were measured during a 5-minute session. The training trials with the Morris water maze were performed from 7 to 11 days after the injection. Escape latency was measured (mean of a group of four trials per day) in the training week (**c**). Results of the probe trial (14 days after the injection) are shown as percentages of time spent in the platform quadrant (**d**) and in the opposite quadrant (**e**). * *p* < 0.05, ** *p* < 0.01, *** *p* < 0.001, comparing the four groups of mice for Y-maze test and probe test; * *p* < 0.05, comparing OLE NaCl and OLE Aβ_42_ groups; ^#^ *p* < 0.05, ^##^ *p* < 0.01, comparing ARA NaCl and ARA Aβ_42_ groups; ^$^ *p* < 0.05, ^$$^ *p* < 0.01, comparing OLE Aβ_42_ and ARA Aβ_42_ groups for training with Morris water maze test. All data are presented as mean ± SEM (*n* = 9–15)

In the Y-maze test, OLE-fed mice injected with the vehicle (*n* = 10) displayed 70.7 ± 2.6% alternation rates, which dropped to 55.2 ± 3.4% when mice were injected with Aβ_42_ oligomers (*n* = 9) (Fig. 3a). Because the number of arm entries did not change significantly between the two subgroups (Fig. 3b), the reduction of alternation rates after injection with Aβ_42_ oligomers (mean reduction 15.5%) was not due to exploratory, locomotor, visual, or motivational effects and indicates an impairment of spatial working memory, as previously reported for BALB/c mice fed a standard diet [15]. ARA-fed mice injected with vehicle (*n* = 14) displayed alternation rates similar to those of OLE-fed mice injected with Aβ_42_ oligomers (53.2 ± 2.8%) (Fig. 3a). Injection with Aβ_42_ oligomers (*n* = 15) did not significantly modify the alternation behavior (49.9 ± 2.1%). Because the number of entries in the ARA subgroups was similar to that in the OLE subgroups, spatial working memory was altered by dietary ARA, and Aβ_42_ oligomers did not amplify this alteration.

In the Morris water maze, OLE- and ARA-fed mice injected with vehicle exhibited the same learning abilities (Fig. 3c). As previously described for standard diet-fed BALB/c mice [15], injection of Aβ_42_ oligomers did not suppress but reduced the learning abilities of the OLE-fed mice (2–9 seconds longer escape latencies across the 5 training days with significant variations for days 2–4) (Fig. 3c). In contrast, injection of the Aβ_42_ oligomers into ARA-fed mice almost completely suppressed their learning abilities (8–14.5 seconds longer escape latencies and significant variations for days 1–5 compared with ARA + NaCl subgroup and for days 4–5 compared with OLE + Aβ subgroup) (Fig. 3c). In the probe test, OLE-fed mice spent less time in the target quadrant and more time in the opposite one when they were given injections of Aβ_42_ oligomers (22% instead of 32.9% and 32% instead of 22.4%, respectively) (Fig. 3d, e). ARA-fed mice trended toward spending less time in the target quadrant (26.7% instead of 32.9% with vehicle and 17.9% instead of 22% with Aβ_42_ oligomers) and more time in the opposite one (25.9% instead of 22.4% with vehicle and 32.9% instead of 32% with Aβ_42_ oligomers) than those that received the OLE diet, regardless of whether they were given injections with vehicle or Aβ_42_ oligomers. However, these differences were not statistically significant (Fig. 3d, e).

### Dietary ARA reduces expression levels of AMPA receptors in absence or presence of Aβ_42_ oligomers

Recently, many studies have established that Aβ_42_ oligomers induced synaptic dysfunction assumed to cause the first memory alterations observed in the early steps of AD [18, 22]. So, we sought to determine if the dietary ARA-induced alterations of spatial working memory and learning abilities could be related to damage of proteins involved in synaptic function. Because AMPA receptors underlie the establishment of LTP and constitute well-known targets of Aβ_42_ oligomers [32, 33], we investigated the effects of dietary ARA on the hippocampal or cortical protein levels of the four members of this family of receptors (GluR1–GluR4) after icv injection of Aβ_42_ oligomers or vehicle.

We first evaluated the expression levels of the AMPA receptors in Western blotting experiments. We found that GluR2 protein levels were reduced by about two-fold by the ARA diet in cortical homogenates, whereas injections of Aβ_42_ oligomers did not further reduce the expression of this AMPA receptor member (Additional file 6). We observed drastic reductions (to 12–20% of the levels observed in the control OLE group after NaCl injections) of the expression levels of the four types of AMPA receptors in the hippocampus homogenates of ARA-fed mice after Aβ_42_ oligomer injections (Additional file 7). The intensities of the Western blot bands corresponding to the various AMPA receptor isoforms appeared as almost wiped out in these homogenates, although such variations could not be strictly correlated to those of the expression levels, owing to lack of linearity of the antibody-antigen response in the Western blot experiments in some ranges of expression.

**Fig. 4:**
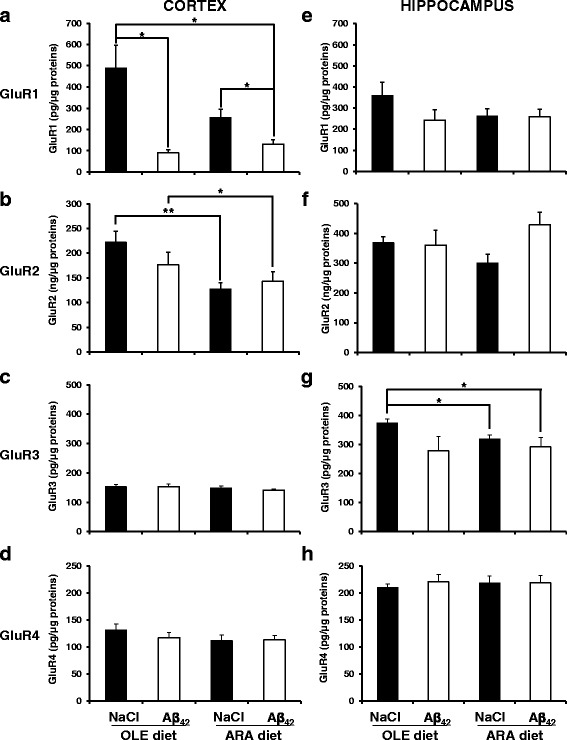
Modification of α-amino-3-hydroxy-5-methyl-4-isoxazolepropionic acid (AMPA) receptors in cortex and hippocampus homogenates induced by arachidonic acid-enriched (ARA) diet and intracerebroventricular injections of amyloid-β peptide 42 oligomers. Immediately after the probe test, mice were killed, and homogenates were prepared from cortex and hippocampus. The expression levels of the four murine members of the AMPA receptor family were measured in cortex (**a**–**d**) and hippocampus **e**–**h** homogenates by using specific enzyme-linked immunosorbent assay (ELISA) kits from Aviva Systems Biology Corporation for glutamate receptor 1 (GluR1) (**a** and **e**) and GluR2 (**b** and **f**) and from Cloud-Clone Corporation for GluR3 (**c** and **g**) and GluR4 (**d** and **h**). Data are expressed as picograms of the specific AMPA receptor family member per microgram of total protein in the brain homogenates (* *p* < 0.05 and ** *p* < 0.01, comparing the four groups of mice). Results are shown as mean ± SEM of ELISA measurements performed for all animals of the group (oleic acid-enriched diet [OLE] groups, *n* = 4; ARA groups, *n* = 6). Measurements were performed in duplicate for each brain tissue sample

To determine if these observations could be due to actual variations of the expression levels of AMPA receptors or alternative technical reasons linked to Western blotting technology, we performed new measurements by using specific ELISA kits for the four AMPA receptor types because ELISA methods allow more quantitative evaluation than the semi-quantitative Western blotting experiments. In cortical homogenates, we found that injections of Aβ_42_ oligomers decreased GluR1 expression levels from 489 ± 106 pg/μg and 258 ± 37 pg/μg total proteins (after NaCl icv injections) to 90 ± 14 pg/μg and 132 ± 21 pg/μg total proteins in the OLE- and ARA-fed mouse groups, respectively (Fig. 4a). The ARA diet reduced GluR2 expression levels in cortical homogenates from 222 ± 22 pg/μg and 176 ± 26 pg/μg total proteins in the OLE-fed mice (after NaCl and Aβ_42_ oligomer injections, respectively) to 128 ± 12 pg/μg and 143 ± 19 pg/μg total proteins in the ARA -fed mice (after NaCl and Aβ_42_ oligomer injections, respectively) (Fig. 4b). No difference could be detected for GluR2 in the cortical homogenates between the mice that received NaCl and Aβ_42_ oligomer icv injections, regardless of the diet used (Fig. 4b). GluR3 and GluR4 expression displayed similar levels in the cortical homogenates from the four mouse subgroups (Fig. 4c and d). In hippocampus homogenates, we could observe that the ARA diet decreased GluR3 expression levels compared with the OLE-fed mice after NaCl icv injection (Fig. 4 g). By contrast, we did not notice any variation of the expression levels of the other AMPA receptors in hippocampus homogenates of the four mouse subgroups, even if there was a trend toward reduction of the GluR1 expression levels in the ARA-fed mice and in the OLE-fed mice after Aβ_42_ oligomer icv injections compared with the control OLE-fed and NaCl icv-injected mouse group (Fig. 1e).

### Dietary ARA reduces expression levels of postsynaptic density protein receptor in cortex

**Fig. 5:**
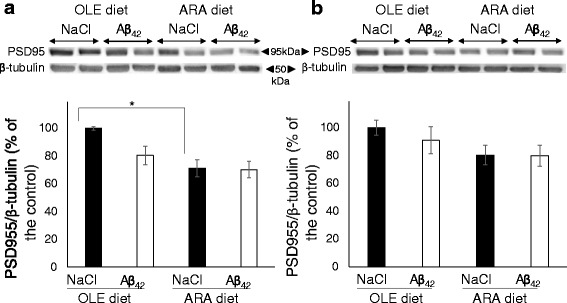
Modification of hippocampal and cortical synaptic postsynaptic density protein 95 (PSD95) induced by arachidonic acid-enriched (ARA) diet. Immediately after the probe test, mice were killed, and synaptosomes were prepared from the cortex and hippocampus. Representative immunoblots of cortical (**a**) and hippocampal (**b**) PSD95 from oleic acid-enriched (OLE) or ARA diet mice after NaCl or amyloid-β peptide (Aβ_42_) injections are shown. Densitometric analyses were performed to determine signal intensities normalized to β-tubulin, and data are expressed as percentages of control OLE mice given injections with NaCl (* *p* < 0.05, comparing the four groups of mice). Results are shown as mean ± SEM of immunoblots performed for all animals (OLE groups, *n* = 4; ARA groups, *n* = 6)

AMPA receptors frequently interact with PSD95 protein complex in synapses to play their role in neurotransmission [34]. Therefore, we examined the effects of two diets on PSD95 protein expression and their modulation by Aβ_42_ oligomers. We observed that injections with Aβ_42_ oligomers induced a mean 20% reduction of PSD95 expression levels in the synaptosomes prepared from the cortex of OLE-fed mice, but this reduction did not reach a statistically significant level (Fig. 5a). In contrast, a significant mean 30% reduction of PSD95 expression level was noted in the cortex of ARA-fed mice given a vehicle injection. Injection with Aβ_42_ oligomers did not further reduce PSD95 expression levels in these mice (Fig. 5a). In the hippocampal synaptosome preparations, roughly similar effects were seen, but with lower mean decreases of 10% and nonsignificant amplitudes (Fig. 5b). No modification in the expression levels of the SNAP25 presynaptic marker was evidenced in the various mouse subgroups (*see* Additional file 8).

To further characterize the mechanism of the influence of dietary ARA on cognitive abilities and underlying synapse function, we first investigated the presence of chronic neuroinflammation that could impair synaptic function and then the change in the balance between cPLA_2_ and neuronal ACSL4, which respectively release ARA from membrane phospholipids and allow its reincorporation into these phospholipids.

### Dietary ARA induces astrocyte proliferation in hippocampus

**Fig. 6:**
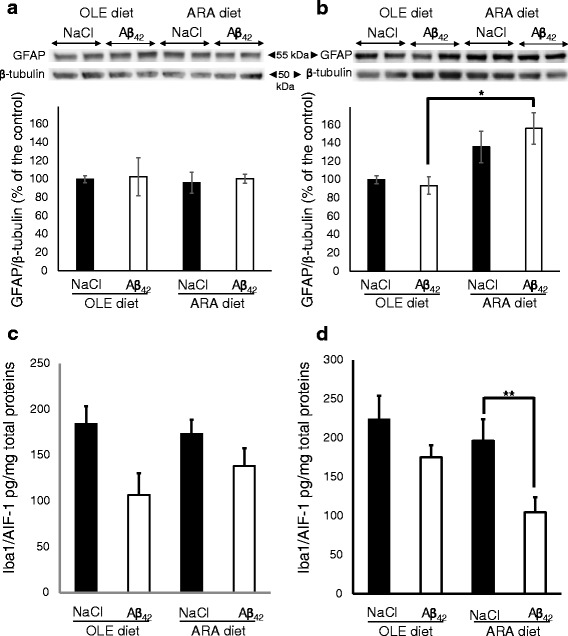
Modifications of brain glial fibrillary acidic protein (GFAP) and Iba1/AIF-1 proteins induced by arachidonic acid-enriched (ARA) diet and amyloid-β peptide 42 (Aβ_42_). Immediately after the probe test, mice were killed, and homogenates were prepared from the cortex and hippocampus. Representative immunoblots of cortical (**a**) and hippocampal (**b**) GFAP astroglial protein from oleic acid-enriched (OLE) or ARA diet mice after NaCl or Aβ_42_ injections are shown. Densitometric analyses were performed to determine signal intensities normalized to β-tubulin, and data are expressed as percentages of control OLE mice injected with NaCl (* *p* < 0.05 and ** *p* <0.01, comparing the four groups of mice). Results are shown as mean ± SEM of immunoblots performed for all animals (OLE groups, *n* = 4; ARA groups, *n* = 6). Expression levels of the Iba1/AIF-1 microglial marker was determined by enzyme-linked immunosorbent assay (ELISA), and the relative amounts of Iba1/AIF-1 per microgram of total protein are shown in the cortex (**c**) and hippocampus (**d**) of the OLE- and ARA-fed mice after intracerebroventricular NaCl or Aβ_42_ injections. Results are shown as mean ± SEM of ELISA measurements in each group of mice (OLE groups, *n* = 4; ARA groups, *n* = 6)

We measured the expression levels of GFAP as a marker of glial proliferation (Fig. a, b). No difference in GFAP levels could be detected in the cortices of the various mouse subgroups, regardless of the diet or the injected solution used (Fig. 6a). In the hippocampus of OLE-fed mice, injection with Aβ_42_ oligomers did not increase the GFAP level. But GFAP expression increased by 1.6-fold in ARA-fed mice after injection with Aβ_42_ oligomers (Fig. 6b). After injection with the vehicle, higher GFAP expression levels were also observed, but the mean increase did not reach statistically significant levels.

Next, we examined whether dietary ARA and/or Aβ_42_ oligomer injections induced microglial activation by using the conventional Iba1/AIF-1 marker. We did not find any increase of the Iba1/AIF-1 expression level in the cortex or hippocampus, regardless of the diet and type of icv injection (Fig. 6c and d). Surprisingly, we observed a reduction of Iba1/AIF-1 expression levels after Aβ_42_ injection in the hippocampus of ARA-fed mice (Fig. 6d). A trend toward reduction of expression levels of Iba1/AIF-1 was also noted in the cortex and hippocampus of the other groups after Aβ_42_ injection, but the level of statistical significance was not reached (Fig. 6c and d). We also used another microglial marker, the TREM-2 triggering receptor, in which upregulation has been observed in AD model mice [35, 36], but we could not find any variation of its expression level in the hippocampus or cortex of our various mice groups (*see* Additional file 9).

### Dietary ARA modulates cPLA_2_ activation and neuronal form of ACSL4

**Fig. 7:**
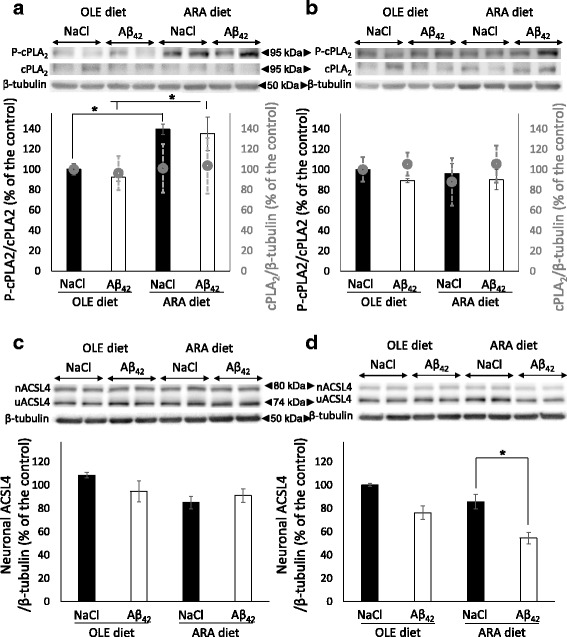
Modifications of brain cytosolic phospholipase A_2_ (cPLA_2_) and neuron-specific acyl-coenzyme A synthetase 4 (nACSL4) proteins induced by arachidonic acid-enriched (ARA) diet and amyloid-β peptide 42 (Aβ_42_). Immediately after the probe test, mice were killed, and homogenates were prepared from the cortex and hippocampus. Representative immunoblots of cortical (**a** and **c**) and hippocampal (**b** and **d**) phosphorylated cytosolic phospholipase A_2_ (P-cPLA_2_) and cPLA_2_ (**a** and **b**) and nACSL4 (**c** and **d**) proteins from oleic acid-enriched (OLE) or ARA diet mice after NaCl or Aβ_42_ injections are shown. Densitometric analyses were performed to determine signal intensities normalized to β-tubulin for cPLA_2_ and nACSL4 and normalized to cPLA_2_ for P-cPLA_2_, and data are expressed as percentages of control OLE mice injected with NaCl (* *p* < 0.05, comparing the four groups of mice). In (**a**) and (**b**), P-cPLA_2_/cPLA_2_ density is shown by the *black* and *white scales* (*y*-axis on the *left*), and cPLA_2_/β-tubulin is shown by the *gray circles* (*y*-axis on the *right*). Results are shown as mean ± SEM of immunoblots performed for all animals (OLE groups, *n* = 4; ARA groups, *n* = 6)

Overexpression or overactivation of the ARA-releasing cPLA_2_ should be associated with increased cell-free ARA levels in the absence of counteracting mechanisms. Free ARA can be reincorporated into membrane phospholipids by the ARA-specific ACSL4, which is expressed as two neuron-specific and ubiquitous isoforms in the brain, as reported above [17]. Therefore, we examined the influence of the two diets and Aβ_42_ oligomer injections on these two ARA-mobilizing enzymes. We first measured cPLA_2_ protein expression and phosphorylation levels on Ser505, which is critical for its activation. We did not observe any modification of cPLA_2_ expression levels in the hippocampus or cortex by dietary ARA or Aβ_42_ oligomers (Fig. 7a and b). In contrast, dietary ARA increased Ser505 phosphorylation level in the cortex but not in the hippocampus, and this effect was maintained but not amplified after Aβ_42_ oligomer injection (Fig. 7a, b).

We next measured the protein levels of the neuron-specific and ubiquitous ACSL4 isoforms in the brain structures of the various groups of mice. No variation in the expression levels of the ACSL4 ubiquitous isoform was observed, but Aβ_42_ oligomer injections reduced by 40% the expression levels of the neuron-specific isoform in the hippocampus of ARA-fed mice (Fig. 7d). This reduction was not observed in the cortex or in the OLE-fed mice (Fig. 7c and d). Then we analyzed phospholipid species to examine whether the variations in expression levels of the neuronal ACSL4 isoform and cPLA_2_ modified ARA incorporation into the cortical and hippocampal phospholipids.

### Modification of phospholipid species profiles by dietary ARA and Aβ_42_ oligomers

Biological effects of ω-6 or ω-3 PUFAs, including on memory, have been associated with the nature of the phospholipids into which they are incorporated as well as their distribution in brain structures [37, 38]. We used liquid chromatography-tandem mass spectrometry (LC-MS/MS) to analyze the phospholipid species putatively affected by ARA diet in the cortex and hippocampus. We focused our analyses on the ARA-, DHA-, DTA-, and docosapentaenoic acid (DPA)-containing species in the hippocampus and cortex of our four subgroups of mice. Our aims were to determine whether dietary ARA intake could modify the equilibrium between ARA- and DHA-containing species in the various glycerophospholipid classes and whether these modifications influence the effects of Aβ_42_ oligomers on specific species and potentially increase the release of ARA. We included the DTA- and DPA-containing species because dietary ARA can be elongated and desaturated into the ω-6 PUFAs DTA and DPA. We measured the areas of ARA-, DTA-, DPA-, and DHA-containing species in each phospholipid class, and we expressed each species as a percentage of the sum of these various areas in each class: phosphatidylcholine (PC), phosphatidylethanolamine (PE), phosphatidylinositol (PI), and phosphatidylserine (PS). To avoid confusion due to isobaric species, we performed MS2 analyses for PI, PE, PS, and MS3 for PC. The data derived from the LC-MS/MS analyses are presented in Table 4.

**Table 4:** Brain phospholipids (percentage of measured phospholipids for each class) among male mice fed oleic acid-enriched or arachidonic acid-enriched diet

Brain structures	Cortex				Hippocampus			
Injections	NaCl		Aβ_42_		NaCl		Aβ_42_	
Diets	OLE	ARA	OLE	ARA	OLE	ARA	OLE	ARA
Phosphatidylinositol								
16:0/20:4	13.10 ± 1.83	13.69 ± 0.33	7.80 ± 0.49^a^	8.28 ± 0.17^a^	10.90 ± 0.49	10.49 ± 0.66	8.08 ± 2.24	7.04 ± 0.21
18:0/20:4	74.30 ± 1.74	76.34 ± 1.37	87.32 ± 1.53^b^	87.22 ± 0.44^b^	78.38 ± 0.56	78.17 ± 0.93	83.32 ± 3.22	83.49 ± 0.54
18:1/20:4	9.27 ± 0.23	7.81 ± 0.97	2.95 ± 1.09^b^	3.06 ± 0.30^a^	9.16 ± 0.32	9.77 ± 0.52	7.61 ± 0.83	7.61 ± 0.42
16:0/22:6	1.07 ± 0.16	0.18 ± 0.04^c^	0.18 ± 0.01^d^	0.22 ± 0.03	0.58 ± 0.02	0.53 ± 0.04	0.48 ± 0.05	0.64 ± 0.01
18:0/22:6	2.26 ± 0.40	1.98 ± 0.25	1.75 ± 0.08	1.22 ± 0.06	0.99 ± 0.09	1.04 ± 0.18	0.51 ± 0.13	1.22 ± 0.74
Arachidonic acid species	96.67 ± 0.57	97.84 ± 0.21	98.07 ± 0.07	98.56 ± 0.06	98.43 ± 0.08	98.42 ± 0.17	99.01 ± 0.17	98.14 ± 0.75
Docosahexaenoic acid species	3.33 ± 0.57	2.16 ± 0.21	1.93 ± 0.07	1.44 ± 0.06	1.57 ± 0.08	1.58 ± 0.17	0.99 ± 0.17	1.86 ± 0.75
Phosphatidylcholine								
16:0/20:4	45.18 ± 4.90	49.97 ± 2.51	45.35 ± 1.57	53.01 ± 2.76	26.94 ± 1.44	28.66 ± 1.42	68.63 ± 1.96^b^	63.14 ± 9.83^a^
18:0/20:4	16.75 ± 3.75	15.2 ± 2.63	27.70 ± 2.36	26,79 ± 1.59	35.68 ± 0.24	37.78 ± 1.18	16.35 ± 2.13^a^	15.05 ± 6.23^b^
18:1/20:4	2.92 ± 0.76	3.38 ± 0.65	5.55 ± 0.95	3.69 ± 0.22	10.29 ± 0.61	10.23 ± 0.71	2.62 ± 0.28^d^	4.83 ± 0.94^b^
18:0/22:6	16.29 ± 1.27	16.30 ± 1.56	1.02 ± 0.07^d^	2.45 ± 0.50^d^	9.05 ± 0.83	7.16 ± 0.57	5.19 ± 0.83	6.81 ± 1.35
18:1/22:6	8.22 ± 1.40	6.80 ± 0.86	0.91 ± 0.15^b^	1.40 ± 0.22^a^	3.04 ± 0.36	2.90 ± 0.05	3.84 ± 0.47	3.10 ± 0.07
16:0/22:6	10.64 ± 3.33	8.43 ± 1.81	19.47 ± 0.25	12.65 ± 1.95	15.00 ± 1.33	13.27 ± 0.37	3.37 ± 1.33^d^	7.07 ± 1.37^a^
Arachidonic acid species	64.85 ± 0.80	68.46 ± 1.04	78.60 ± 0.15^d^	83.49 ± 1.45^d^	72.91 ± 2.09	76.67 ± 0.97	87.61 ± 0.14^b^	83.02 ± 2.66
Docosahexaenoic acid species	35.15 ± 0.80	31.54 ± 1.04	21.40 ± 0.15^d^	16.51 ± 1.45^d,e^	27.09 ± 2.09	23.33 ± 0.97	12,39 ± 0.14^b^	16.98 ± 2.66
Phosphatidylserine								
18:0/20:4	3.18 ± 0.26	11.16 ± 0.35^c^	1.47 ± 0.08^b^	1,92 ± 0.07^d^	6.17 ± 0.23	7.12 ± 0.40	3.70 ± 0.16^b^	5.36 ± 0.37^a,e^
16:0/22:4	0.13 ± 0.02	0.17 ± 0.01	0.21 ± 0.01	0.22 ± 0.03	0.21 ± 0.02	0.26 ± 0.03	0.077 ± 0.010^b^	0.093 ± 0.009^b^
18:0/22:4	6.35 ± 0.26	9.11 ± 0.18^c^	5.41 ± 0.14	9.10 ± 0.35^c^	7.73 ± 0.36	10.57 ± 0.03	6.31 ± 0.32	10.75 ± 1.35^e^
18:1/22:5	0.43 ± 0.07	0.46 ± 0.01	0.38 ± 0.08	0,45 ± 0.02	0.41 ± 0.02	0.48 ± 0.06	0,41 ± 0.04	0.44 ± 0.01
18:0/22:6	89.91 ± 0.40	79.10 ± 0.53^c^	92.53 ± 0.19^a^	88.32 ± 0.40^c,d^	85.49 ± 0.58	81.57 ± 0.43	89.50 ± 0.46	83.35 ± 1.69^e^
Phosphatidylethanolamine								
a16:0/20:4	3.23 ± 0.04	4.04 ± 0.07	1.70 ± 0.24^b^	2.80 ± 0.16^b,e^	3.51 ± 0.09	4.17 ± 0.15	2.74 ± 0.13	4,54 ± 0.99
p16:0/20:4	3.58 ± 0.19	4.02 ± 0.04	1.06 ± 0.01^d^	2.39 ± 0.14^c,d^	2.41 ± 0.28	2.39 ± 0.16	1.59 ± 0.08	2,67 ± 0.51
a18:0/20:4	18.29 ± 1.66	16.97 ± 0.26	18.03 ± 0.16	19.38 ± 0.44	20.52 ± 0.36	21.31 ± 0.96	22.66 ± 0.65	13.57 ± 5.29
p18:0/20:4	4.70 ± 0.19	5.78 ± 0.24	5.12 ± 0.24	6.43 ± 0.09^e^	4.45 ± 0.31	5.02 ± 0.30	4.93 ± 0.19	8.68 ± 1.91
a18:1/20:4	2.22 ± 0.11	2.38 ± 0.06	0.41 ± 0.09^d^	0.84 ± 0.08^d,e^	2.47 ± 0.06	2.84 ± 0.03	1.18 ± 0.07^b^	1.17 ± 0.36^b^
p18:1/20:4	2.79 ± 0.18	3.20 ± 0.08	1.21 ± 0.23^b^	2.13 ± 0.10^b,e^	2.50 ± 0.17	2.87 ± 0.21		4.22 ± 0.95
a18:2/20:4	0.03 ± 0.01	0.04 ± 0.005	0.02 ± 0.001	0.02 ± 0.003	0	0	0.025 ± 0.001^b^	0.014 ± 0.010
p18:2/20:4	0.04 ± 0.004	0.03 ± 0.004	0.02 ± 0.004	0.02 ± 0.002	0.048 ± 0.007	0	0.032 ± 0.004^b^	0.031 ± 0.008^a^
p16:0/22:4	3.15 ± 0.14	3.76 ± 0.15	2,91 ± 0.24	4,29 ± 0.08^f^	3.12 ± 0.33	3.71 ± 0.13	3.67 ± 0.05	6.13 ± 1.29
p16:1/22:4	0	0.01 ± 0.00	0.0015 ± 0.0007	0.0029 ± 0.0003^b^	0	0	0.0033 ± 0.0005	0,006 ± 0.0016^a^
a18:0/22:4	3.97 ± 0.41	4.51 ± 0.24	1.55 ± 0.06^d^	2.25 ± 0.11^b^	3.02 ± 0.01	3.78 ± 0.29	1.94 ± 0.11	4.34 ± 0.71^e^
a18:1/22:4	0.21 ± 0.01	0.26 ± 0.01^e^	0.10 ± 0.01^d^	0.20 ± 0.002^a,c^	0.22 ± 0.06	0.28 ± 0.01	0.17 ± 0.01	0,35 ± 0.08^e^
a18:0/22:5	7.13 ± 0.57	7.20 ± 0.09	5.19 ± 0.21^a^	5.85 ± 0.12	4.47 ± 0.15	5.23 ± 0.10	5.26 ± 0.09	6,67 ± 1.70
a16:0/22:6	7.10 ± 0.14	6.24 ± 0.06^f^	4.03 ± 0.06^d^	3.71 ± 0.06^d^	5.89 ± 0.01	5.49 ± 0.24	3.85 ± 0.05	1.12 ± 0.80^b,e^
p16:0/22:6	6.02 ± 0.15	4.69 ± 0.14^f^	3.62 ± 0.17^d^	2.74 ± 0.06^d,e^	4.90 ± 0.40	5.46 ± 0.09	4.29 ± 0.21	4,90 ± 0.72
a18:0/22:6	28.25 ± 3.25	22.01 ± 0.55	25.14 ± 0.42	22.31 ± 0.06	20.70 ± 0.42	17.94 ± 0.37	22.28 ± 0.27	23.27 ± 6.20
p18:0/22:6	11.76 ± 1.53	10.31 ± 0.51	16,14 ± 0.46	13.10 ± 0.61	16.72 ± 1.60	14.60 ± 0.99	17.65 ± 1.01	9,16 ± 3.54
a18:1/22:6	2.03 ± 0.29	1.36 ± 0.05	12.66 ± 1.05^d^	10.33 ± 0.79^d^	1.55 ± 0.16	1.27 ± 0.04	5,32 ± 1.00^b^	1,18 ± 0.23^f^
p18:1/22:6	3.34 ± 0.13	3.19 ± 0.16	1.11 ± 0.23^d^	1.19 ± 0.08^d^	4.13 ± 0.31	3.64 ± 0.22	2,43 ± 0.37	1.61 ± 0.55^a^
Arachidonic acid species	30.11 ± 3.0	36.46 ± 0.09	27.56 ± 0.94	34.02 ± 0.69	35.92 ± 0.44	38.60 ± 1.27	33,16 ± 0.32	41.28 ± 6.36
Docosahexaenoic acid species	56.49 ± 3.17	47.80 ± 0.24^e^	62,69 ± 0.93	53,39 ± 0.73^e^	53.25 ± 0.95	48.40 ± 1.35	55.81 ± 0.33	41.23 ± 2.85^f^
Docosatetraenoic acid species	6.27 ± 0.64	8.53 ± 0.11^e^	4,56 ± 0.21	6.74 ± 0.16^a,e^	6.36 ± 0.36	7.77 ± 0.27	5.78 ± 0.08	10.83 ± 2.07
Docosapentaenoic acid species	7.13 ± 0.57	7.20 ± 0.09	5,19 ± 0.21^a^	5.85 ± 0.12	4.47 ± 0.15	5.23 ± 0.10	5.26 ± 0.09	6.67 ± 1.70

*Aβ*_*42*_ Amyloid-β peptide 42, *ARA* Arachidonic acid-enriched diet, *OLE* Oleic acid-enriched diet

^a^ *p* < 0.05, Aβ_42_ compared with NaCl

^b^ *p* < 0.01, Aβ_42_ compared with NaCl

^c^ *p* < 0.001, ARA compared with OLE

^d^ *p* < 0.001, Aβ_42_ compared with NaCl

^e^ *p* < 0.05, ARA compared with OLE

^f^ *p* < 0.01, ARA compared with OLE

Several differences were observed between ARA- and OLE-fed mice in some PS and PE species and in only one PI species in the cortex. As previously reported [39, 40], ARA-containing species predominate in PI in the cortex as well as in the hippocampus, representing 96–99% of the ARA- and DHA-containing species. The minor PI species 16:0/22:6 was reduced by 83% in the cortex (from 1.07 ± 0.16% to 0.18 ± 0.04%) by the ARA diet compared with the OLE diet. The icv injections of Aβ_42_ oligomers collapsed this species to 17% of its initial level in OLE-fed mice (i.e., the same level as in NaCl- or Aβ_42_ oligomer-injected mice fed the ARA diet) (Table 4). The icv injections of Aβ_42_ oligomers also shifted the distribution of ARA from the two minor ARA-containing PI species, 16:0/20:4 and 18:1/20:4, to the major one, 18:0/20:4, in the cortex without any difference between OLE- and ARA-fed mice. The most numerous differences in cortical phospholipids between the OLE- and ARA-fed mice were observed in the PS class. The ARA diet increased the ω-6 18:0/20:4 and 18:0/22:4 PS species of mice given icv NaCl injections by 3.5 and 1.4, respectively, whereas it reduced by 12% the ω-6 18:0/22:6 species (Table 4). Interestingly, the injection of Aβ_42_ oligomer reduced the 18:0/20:4 species in ARA-fed mice to the same levels as in OLE- and ARA-fed mice, suggesting that the release of ARA was higher in ARA-fed mice in the presence of Aβ_42_ oligomers than in OLE-fed mice, because their initial ARA amounts were higher. By contrast, the DTA-containing species 18:0/22:4 was not affected by icv injections of Aβ_42_ oligomers, and the relative levels of the DHA-containing 18:0/22:6 species were increased (Table 4). In cortical PE species, only the acyl a16:0/22:6 and plasmalogen p16:0/22:6 species showed a significant decrease in ARA-fed mice compared with the OLE-fed mice, whereas the minor a18:1/22:4 species was slightly increased and the various ARA species did not differ between the two groups of mice. Aβ_42_ oligomer icv injections reduced several ARA- and DTA-containing species. In addition, they also decreased the levels of several DHA-containing species, but they increased by 6.5- to 7.5-fold the a18:1/22:6 species. No clear difference was observed between the ARA- and OLE-fed mice that received with Aβ_42_ oligomer injections. Similarly, the icv injections of Aβ_42_ oligomers collapsed the 18:0/22:6 and 18:1/22:6 PC species (reduction by 5- to 16-fold), but without any difference between the ARA- and OLE-fed mice (Table 4).

The differences observed in the cortical PI, PS, and PE phospholipid classes between OLE- and ARA-fed mice after NaCl injections were not found in the hippocampus (Table 4). By contrast, icv Aβ_42_ oligomer injections induced modifications of several species in PS, PC, and PE classes, but not in PI, in the hippocampus. Several differences between ARA- and OLE-fed mice were observed in hippocampal PS classes after icv Aβ42 oligomer injections. However, the levels of statistical significance of these differences were lower than in the cortex, and they more frequently affected DTA- and DHA-containing species than ARA-containing species. Only the PS 18:0/20:4 species differed between OLE- and ARA-fed mice after Aβ_42_ oligomer injection, but they were higher in the hippocampus of ARA-fed mice instead of being lower as in the cortex (Table 4). Finally, many specific DHA-containing PE species and their global sum were reduced in Aβ_42_ oligomer-injected and ARA-fed mice compared with Aβ_42_ oligomer-injected and OLE-fed mice. To summarize, these phospholipid species analyses indicated that the ARA diet favors ARA incorporation and release by Aβ_42_ oligomers in the cortex, mainly in the PS class, whereas no clear evidence of this was obtained in the hippocampus, although many modifications were observed in PE and PS DTA- or DHA-containing species.

## Discussion

### Acute icv administration of Aβ_42_ peptide oligomers to mice as AD murine model to study environmental influence on their neurotoxicity

In this study, we used an AD model consisting in a single icv injection of 500 pmol Aβ_42_ peptide oligomers. In the transgenic murine models that are used in most studies about AD and the role of Aβ peptides, the high and chronic production of these peptides prevents consideration of the environmental influences separately on their production, their aggregation, or the sensitivity to their neurotoxicity. On the contrary, the localized administration of a known amount of Aβ_42_ peptide oligomers allows analysis of the sensitivity to Aβ_42_ oligomers of animals preexposed to environmental parameters such as nutritional components during a determined period, which can be useful in the design of preventive strategies in humans. This kind of model also constitutes an approach to examining the early AD steps instead of familial and chronic exposures as in the case of the murine transgenic model expressing mutant human *APP* or presenilin genes. Our oligomer preparations do not contain fibrils, but they contain a mixture of monomers and low-molecular-weight oligomers, as described in the Methods section above. The content in Aβ_42_ monomers must also be taken into account because these monomers are considered nontoxic molecules [41]. We originally showed that a single injection of 500 pmol Aβ_42_ peptide oligomers induced alterations of cognitive abilities in the Y-maze and Morris water maze tests in a 2-week period after injection [25]. We further determined that Aβ_42_ oligomer administration led to synaptosomal reductions of synaptic protein levels and alterations of the survival Janus kinase/signal transducer and activator of transcription and Akt transduction pathways [15, 26]. Many researchers have described such stereotactic administration of Aβ peptides, although the number of injections or infusions, the targeted region, the concentration and the type of Aβ peptides used differed. For example, Moroni et al. [42] recently reported synaptic alterations and memory dysfunction associated with oxidative stress and transduction pathway modifications by icv injection of Aβ_42_ peptide oligomers in the same range of concentrations that we used. The very fast clearance of injected Aβ_40_ peptide and its local degradation were recently reported by McIntee et al. [43], who suggested that Aβ_42_ oligomers used in their study induced deleterious effects shortly after injection and could not be detected after the behavioral experiments. The rationale of the use of stereotactic Aβ_42_ oligomer administration to the mouse brain as an AD model is based on these direct effects on synaptic homeostasis and neuroinflammation [44, 45].

### Previous studies on ARA influence in AD

To our knowledge, only two teams have examined the influence of dietary ARA in AD, and they found opposite results regarding Aβ production in AD murine transgenic models [46–48]. One group [46] observed higher Aβ_42_ production and deposition by feeding young CRND8 mice (3 weeks old) for 21 weeks with high amounts of ARA (2% with 1% soybean oil). The second team [47, 48] administrated low amounts of ARA (0.22%) in a 5% lipid diet to 5-month-old Tg2576 mice at the beginning of the experiments. The latter group observed attenuation of memory impairments after administering a 4-month diet [47] and reductions of Aβ production and deposition after 12 months [48]. These experiments emphasize the fact that opposite results can be obtained by using different AD models and diet protocols. Indeed, it is notable that the CNRD8 line bearing a double mutation of APP displays amyloid plaques at the age of 3 weeks instead of 1 year in the Tg2576 line.

We previously provided evidence of the putative influence of ARA release in the brain on the neurotoxicity of Aβ oligomers by showing that BALB/c cPLA_2_^−/−^ mice are fully resistant to these AD agents [15]. In this former study, we used the same AD model consisting of a single injection of 0.5 nmol of Aβ oligomers before testing the cognitive abilities in Y-maze and Morris water maze tests in the following 2 weeks [15, 25]. The BALB/c male cPLA_2_^−/−^ group was fed the same standard diet that was given to the mice during the habituation period in the present study. As cited above, cPLA_2_ specifically releases ARA [12] and is activated by Aβ oligomers in neuronal cells [13–15]. Therefore, all the events that increase ARA availability in the brain and/or cPLA_2_ activity could increase Aβ oligomer neurotoxicity. In the present study, we investigated if the increase of ARA dietary intake leads to the enhancement of ARA incorporation in the brain and sensitivity to Aβ oligomers, and we explored putative mechanisms. Supplementation with fatty acids or other molecules of interest are frequently used in nutritional studies. The drawback of such approaches is that the control diet is not strictly equivalent and provides less lipid and energy than the tested diet. Therefore, we used two diets containing higher amount of lipids than the standard diets found on the market to compensate ARA by another fatty acid (i.e., monounsaturated OLE). We chose OLE instead of the ω-3 or saturated fatty acids because these latter ones have clear physiological effects on health, mainly in the cardiovascular system.

### ARA and OLE diets: comparison with conventional and high-fat diets

Several studies underline the relationship between AD and obesity as well as the influence of high-fat diets [49]. Our ARA-enriched diet as well as the OLE-enriched control diet provided 23% energy as lipid intake instead of 15% energy with standard diets. High-fat diets used in many studies provide up to 60% of energy as fat intake [50]. The fact that our OLE and ARA diets cannot be considered high-fat diets is confirmed by the fact that the body mass of our mice was similar to those previously reported for young (4-week-old) BALB/c male mice fed a standard diet for 9 weeks, and our mice showed moderate body mass increase, less than 6 g in 12 weeks instead of 21.5 g in 7 weeks in male mice fed a high-fat diet [51]. In addition, we did not observe any basal hypercholesterolemia or hypertriglyceridemia in our various groups of mice. However, the ARA diet slightly decreased postprandial hypertriglyceridemia, which has not been reported until now, to our knowledge.

Our ARA and OLE diets provided more lipids (10% instead of 6.2%) and different amounts of various fatty acids than the standard diet, which was given to the mice before the experiments and is similar to many conventional diets (*see* Table 1). Conventional rodent diets frequently contain around 4–5% lipids and high amounts of linoleic acid compared with the other fatty acids. Modifications of composition of erythrocyte fatty acids such as OLE, ARA, and linoleic acid are consistent with the variations of dietary intakes of several fatty acids in OLE and ARA diets compared with standard diets. However, some modifications were not expected. Indeed, the OLE and ARA diets provided similar amounts of saturated fatty acids, which were higher than those found in the standard diet, but palmitic acid amounts decreased in erythrocytes with both the ARA and OLE diets, and the ARA diet reduced stearic acid in erythrocytes and the liver compared with the OLE diet. Similarly, the OLE diet increased palmitoleic acid amounts in erythrocytes and in the liver compared with standard (for erythrocytes) and ARA (for erythrocytes and liver) diets, which suggests that Δ9-desaturase uses palmitic acid more efficiently as a substrate in the presence of higher amounts of OLE. The OLE diet, which provided higher amounts of α-linolenic acid and DHA than the standard diet but no EPA, led to EPA and DHA enrichment of erythrocytes, whereas α-linolenic acid remained constant. By contrast, the ARA diet, which contained amounts of DHA and α-linolenic acid similar to those found in the OLE diet, reduced DHA amounts in erythrocytes and led to lower amounts of EPA and DHA in these cells. These results on ω-3 fatty acids suggest that dietary α-linolenic acid is efficiently converted into DHA and EPA in the presence of higher intake of OLE, whereas dietary ARA competes with incorporation of EPA and DHA. Because the ARA diet did not contain higher amounts of α-linoleic acid than the OLE diet, this competition cannot occur at the level of the Δ6-/Δ5-desaturases and elongases, but it can involve transporters or enzymes regulating incorporation of fatty acids into phospholipids and triglycerides such as ACSLs and lysophospholipid acyltransferases. Finally, dietary ARA is likely to be converted into DTA because DTA amounts increased in erythrocytes and the liver of ARA-fed mice.

The large differences we observed between the fatty acid composition of ARA- and OLE-fed mice in peripheral tissues were restricted to OLE, ARA, DTA, and DHA as well as, to a much lower extent, in the cortex and hippocampus. Especially in the hippocampus, no competition between ARA and DHA was found in GC analyses of fatty acid composition or in phospholipid species analyzed by LC-MS/MS. This underlines that brain lipid homeostasis is strictly regulated and that the mechanisms of fatty acid transportation through the blood-brain barrier and incorporation into brain cells play a critical role and should be studied more extensively.

### Effects of dietary ARA on cognitive ability and synaptic proteins

In a former study [15], we showed that the BABL/c male mice fed the standard diet exhibited drastic reductions in short-term memory and learning abilities after icv injections of Aβ_42_ oligomers. These memory alterations were similar to those found in the present study for the OLE-fed mice. Those observed in the ARA-fed mice differed in two respects. First, ARA-fed mice displayed lower short-term memory abilities even after injection of saline control solution. Because the alternation rate was close to the value obtained in a random situation, it was not possible to detect if Aβ_42_ oligomers further reduced the short-term memory abilities in the Y-maze test. Second, learning abilities were clearly more sensitive to icv Aβ_42_ oligomer injections in ARA-fed mice.

AD is considered by several authors as a synaptic disease, at least during its early steps, and several studies indicate that Aβ oligomers inhibit the LTP, which leads to alterations of synaptic function and remodeling of the synaptic network [52]. The action of Aβ oligomers on LTP is mediated through the potentiation of extrasynaptic NMDA receptors and consecutive calcium influx, which is associated with a loss of AMPA receptors at the synapse [53, 54]. In addition, researchers in several studies reported that loss of memory is associated with reductions of expression of AMPA receptor family members [55]. Therefore, we investigated the relationship between the OLE and ARA diets and the expression levels of these receptors in Western blotting and ELISA experiments. In the cortex, both methods showed that the ARA diet decreased GluR2 expression levels, whereas only ELISA evidenced a negative and diet-independent effect of Aβ_42_ oligomer injections on GluR1, this last result being consistent with the previous report by Wakabayashi et al. on decreased GluR1 expression in the cortex of patients with AD [56]. The failure of the Western blotting experiments to show the effect of Aβ_42_ oligomer on GluR1 expression could be due to the large experimental variations and consequent inability to obtain significant differences between the various subgroups. In the hippocampus, only a negative influence of ARA diet on GluR3 expression was shown by ELISA experiments, which did not reproduce our Western blotting data showing drastic reductions of expression levels of the four AMPA receptors (up to 90%) by the combination of ARA diet with Aβ_42_ oligomer administration. These Western blotting experiments were reproduced several times, and we used the same samples with both measurement methods. Furthermore, the primary antibodies used in the ELISA kits or Western blot experiments do not target the same epitopes in the various AMPA receptors according to the furnisher’s information. We assume that posttranslational modifications of AMPA receptors could have interfered with the protein transfers or antibody recognition in the Western blot experiments. It is noteworthy that activation of AMPA receptor phosphorylation and modification of the agonist binding by lipoxygenase metabolites of ARA have been reported by several study groups. In addition, other posttranslational modifications affect AMPA receptors, such as ubiquitinylation or palmitoylation, which could modify their recognition by antibodies in biological samples [57]. Reduced expression of several AMPA receptors has been reported previously in aging [58] or in rodent models of neurological disorders such as depression [59] or epilepsy [60]. However, the mechanisms supporting these modifications have not been determined, although neurotrophin receptor-associated transduction pathways could control AMPA receptor expression [61]. Understanding of the link between the AMPA receptor regulations and the degradation of memory abilities induced by the ARA diet and Aβ_42_ oligomers requires additional exploration.

In our former study [15], alterations of memory abilities were associated with drastic reductions of expression of several pre- and postsynaptic markers in the hippocampus, but those of AMPA receptor were not investigated [15]. In the present study, we found evidence of a nonsignificant decrease of PSD95 expression levels in the cortex and hippocampus of OLE-fed mice after Aβ_42_ oligomer icv injections, and expression levels of the presynaptic marker SNAP25 were not altered. In addition, ARA-fed mice displayed a moderate cortical reduction of PSD95 expression levels in the absence of Aβ_42_ oligomers, and Aβ_42_ oligomers did not induce further reduction. Therefore, we cannot exclude that dietary fatty acids present in higher amounts in OLE and ARA diets than in standard diets exert some protective effects on these markers, if not on cognitive capacity. DHA is the first candidate because both the OLE and ARA diets provided higher amounts of this well-known protective fatty acid than the standard diet did. OLE is the second candidate because the ARA diet contained 27.3% of this fatty acid instead of 18.4% as in the standard diet. However, although Amtul et al. [62] showed that OLE decreased Aβ production in a murine AD model, researchers in other studies reported overexpression of the OLE-synthetizing enzyme stearoyl-CoA desaturase in the brains of patients with AD [63]. These conflicting reports require additional studies to establish a putative neuroprotective role for OLE.

### Dietary ARA intake and neuroinflammation

Several studies have suggested that increase of dietary ARA intake or tissue ARA amounts favors the occurrence of systemic chronic inflammation [64] and the severity of pathologies with a strong inflammatory component, such as chronic inflammatory intestinal diseases [65], arthritis [66], and atherosclerosis [67]. This effect would be mediated by the increase of cellular free ARA and its conversion into proinflammatory eicosanoids, especially in individuals with some genetic backgrounds and in animal models [68]. Furthermore, neuroinflammation is one of the pathological components of AD, and researchers in many studies have reported higher expression of enzymes using ARA as a substrate, such as cyclooxygenases and prostaglandin synthases [69, 70], or brain production of proinflammatory eicosanoids in an AD murine model [14]. However, despite the fact that neuroinflammation can induce the first synaptic dysfunction and cognitive impairment in AD, before Aβ peptide accumulation and plaque formation [71, 72], it is not known if higher ARA levels in the brain facilitate neuroinflammation. We found a moderate increase of GFAP expression in the hippocampus of ARA-fed mice without any evidence of activation of microglial cells. Surprisingly, we observed even a slight decrease of the microglial marker Iba1 in the ARA-fed mice given Aβ_42_ oligomer injections. However, we previously did not observe any GFAP or Iba1 overexpression in the cortex or hippocampus of male BALB/c mice 2 weeks after icv Aβ_42_ oligomer injections in our former study [15]. Activation of glial cells is an early reaction to Aβ oligomers. Balducci et al. showed that icv injections of 7.5 pmol Aβ_42_ oligomers induced Iba1 overexpression before 8 h after injection; this overexpression disappeared after 24 h, whereas GFAP overexpression was maintained [73]. In addition, clearance and degradation of Aβ peptide seems to be very fast after a single icv injection [43]. Therefore, despite the fact that we injected higher amounts of Aβ_42_ oligomers, we cannot exclude that the hippocampal GFAP overexpression that we observed in ARA diet-fed mice after icv Aβ_42_ oligomer injection was not the remaining sign of a final inflammation phase. Additional experiments on inflammation kinetics in our murine model and using various diets would be necessary to clarify this point.

### Role of enzymes cPLA_2_ and ACSL4 using ARA as substrate

The combination of cPLA_2_ and ACSL4 activities with those of unidentified lysophospholipid acyltransferases allows ARA mobilization and transfer between its free form and several phospholipid species according the Lands cycle [16]. Our experiments showed that the cPLA_2_ activation level is higher in the cortex of ARA-fed mice, but not in the hippocampus, although learning impairments and reductions of AMPA receptor expression indicate that the hippocampus should be the target structure of ARA-induced modifications. Interestingly, PC is the cPLA_2_ favorite substrate, and LC-MS/MS analyses showed that the effects of Aβ_42_ oligomers on PC species differ in the cortex. However, differences of phospholipid species between ARA- and OLE-fed mice were preferentially observed in PS and PE classes and affected specific ARA or DHA species. In addition, the 18:0/20:4 PS species was also affected by Aβ_42_ oligomers and can be considered as a putative source of free ARA in the presence of this neurotoxic agent. Therefore, the various modifications of phospholipid species observed in the cortex cannot be related only to cPLA_2_ activity and are the result of complex remodeling mechanisms involving several enzymes and reaction steps. Furthermore, LC-MS/MS analyses confirmed the GC measurements regarding the effect of dietary ARA on DTA, the amount of which was increased in PS and PE species, probably through the elongation of ARA.

Expression of the neuronal isoform of ACSL4 was reduced in the hippocampus of ARA-fed mice after icv Aβ_42_ oligomer injections, but LC-MS/MS analyses did not provide evidence of drastic differences in hippocampal phospholipid species between ARA- and OLE-fed mice. The authors who identified this neuron-specific ACSL4 isoform assumed that it plays a role in neuronal differentiation of stem cells, but its putative function in adult neurons has not been characterized until now. ARA is involved in vesicle trafficking and fusion of vesicles to membranes through the formation of a TIP30 complex that associates TIP30 endophilin B with ACSL4 [74]. In this complex, TIP30 transfers ACSL4-produced arachidonoyl-CoA onto phosphatidic acid, forming new species able to induce close contact between membranes. It is possible that the reduction of neuronal ACSL4 expression and the consequent deficit in arachidonoyl-CoA inhibit the formation of phosphatidic acid species and fusion of the AMPA receptors, merging vesicles with the synaptic membranes. This would result in the orientation of these vesicles with their AMPA receptors toward the lysosomal degradation pathway [75]. However, expression levels of GluR3 decreased in the hippocampus (and in the cortex for GluR2) in ARA-fed mice that were given saline solution injections. This result can be linked to the alteration of working memory in these mice. Because ACSL4 expression is not significantly altered in this group of mice, the effect of ARA should also be mediated by an ACSL4-independent mechanism. The relationship between reductions of AMPA receptor expression levels, ARA mobilization, and ACSL4 activity requires further study to be clearly established.

Variations of the activity or expression of proteins involved in the mobilization of ARA could lead to different biological effects of ARA for the same food intake. In this sense, although our work clearly shows that excessive dietary ARA intake can have deleterious effects on sensitivity to Aβ_42_ oligomer neurotoxicity, it has some limitations, and additional experiments are necessary to determine whether various factors such as the age or the strain of the tested mice could modify ARA incorporation and metabolism.

## Conclusions

Our results suggest that an excess of ARA in the human diet could constitute a risk factor for neurodegenerative diseases such as AD. The cellular basis of the increase in the deleterious effects of the Aβ_42_ oligomers on the mice’s memory induced by dietary ARA requires further investigation to be fully elucidated, but it appears to be at least partially linked to the ARA and DHA composition of neuronal phospholipid membranes and to the enzymes involved in ARA mobilization. Studies of these enzymes in several populations and of the ARA composition of the human diet should be done to confirm this new risk factor and to improve preventive measures against AD.

## Additional files

Additional file 1: Composition of the Aβ_42_ oligomer preparations. The Aβ_42_ oligomer preparation was separated by electrophoresis on nondenaturating 10% polyacrylamide gels. Monomers (4.6 kDa), dimers (9.2 kDa), trimers (13.8 kDa), and tetramers (18.5 kDa) were observed after Coomassie Blue staining. (PPTX 308 kb)

Additional file 2: Cholesterolemia levels after oleic acid-enriched (OLE) and arachidonic acid-enriched (ARA) diets. Body weight postprandial and 6-h fasting plasma cholesterol levels were measured by ELISA after 12 weeks of ARA or OLE diets. Data are expressed in grams per liter (*n* = 16 per group). All data are presented as mean ± standard error of the mean (SEM). (PPTX 64 kb)

Additional file 3: Liver fatty acid composition in the four mouse subgroups (diets and nature of icv injections): OLE diet + NaCl injection, OLE diet + Aβ_42_ oligomer injection, ARA diet + NaCl injection, and ARA diet + Aβ_42_ oligomer injection. (DOCX 19 kb)

Additional file 4: Erythrocyte fatty acid composition in the four mouse subgroups (diets and nature of icv injections): OLE diet + NaCl injection, OLE diet + Aβ_42_ oligomer injection, ARA diet + NaCl injection, and ARA diet + Aβ_42_ oligomer injection. (DOCX 19 kb)

Additional file 5: Cortex and hippocampus fatty acid composition in the four mouse subgroups (diets and nature of icv injections): OLE diet + NaCl injection, OLE diet + Aβ_42_ oligomer injection, ARA diet + NaCl injection, and ARA diet + Aβ_42_ oligomer injection. (DOCX 18 kb)

Additional file 6: Modification of cortical AMPA receptors induced by ARA-enriched diet. Immediately after the probe test, mice were killed, and homogenates were prepared from the cortex. Representative immunoblots of cortical GluR1 (**a**), GluR2 (**b**), GluR3 (**c**), and GluR4 (**d**) from OLE or ARA mice after NaCl or Aβ_42_ injections are shown. Densitometric analyses were performed to determine signal intensities normalized to β-tubulin. Data are expressed as the percentage of control OLE mice injected with NaCl (* *p* < 0.05 comparing the four groups of mice). Results are shown as mean ± SEM of immunoblots performed for all animals (OLE groups *n* = 4, ARA groups *n* = 6). (PPTX 252 kb)

Additional file 7: Modification of hippocampal AMPA receptors induced by ARA diet and Aβ_42_. Immediately after the probe test, mice were killed, and homogenates were prepared from the hippocampus. Representative immunoblots of hippocampal GluR1 (**a**), GluR2 (**b**), GluR3 (**c**), and GluR4 (**d**) from OLE or ARA mice after NaCl or Aβ_42_ injections are shown. Densitometric analyses were performed to determine signal intensities normalized to β-tubulin. Data are expressed as the percentage of control OLE mice injected with NaCl (* *p* < 0.05 comparing the four groups of mice). Results are shown as mean ± SEM of immunoblots performed for all animals (OLE groups *n* = 4, ARA groups *n* = 6). (PPTX 253 kb)

Additional file 8: Modification of hippocampal and cortical presynaptic protein SNAP25 expression induced by ARA diet. Immediately after the probe test, mice were killed, and synaptosomes were prepared from the cortex and hippocampus. Representative immunoblots of cortical (**a**) and hippocampal (**b**) SNAP25 from OLE or ARA mice after NaCl or Aβ_42_ injections are shown. Densitometric analyses were performed to determine signal intensities normalized to β-tubulin, and data are expressed as the percentage of control OLE mice injected with NaCl. Results are shown as mean ± SEM of immunoblots performed for all animals (OLE groups *n* = 2, *n* = 4; ARA groups *n* = 2, *n* = 6). (PPTX 59 kb)

Additional file 9: Modifications of brain TREM-2 proteins induced by ARA diet and Aβ_42_. Immediately after the probe test, mice were killed, and homogenates were prepared from the cortex and hippocampus. Representative immunoblots of cortical (**a**) and hippocampal (**b**) TREM-2 glial proteins from OLE or ARA mice after NaCl or Aβ_42_ injections are shown. Densitometric analyses were performed to determine signal intensities normalized to β-tubulin, and data are expressed as the percentage of control OLE mice injected with NaCl. Results are shown as mean ± SEM of immunoblots performed for all animals (OLE groups *n* = 4, ARA groups *n* = 6). (PPTX 1029 kb)

## Abbreviations

Aβ: Amyloid-β peptide
ACSL4: Acyl-coenzyme A synthetase 4
AD: Alzheimer’s disease
AMPA: α-Amino-3-hydroxy-5-methyl-4-isoxazolepropionic acid
ARA: Arachidonic acid
CoA: Coenzyme A
cPLA_2_: Cytosolic phospholipase A_2_
DHA: Docosahexaenoic acid
DPA: Docosapentaenoic acid
DTA: Docosatetraenoic acid
EDTA: Ethylenediaminetetraacetic acid
ELISA: Enzyme-linked immunosorbent assay
EPA: Eicosapentaenoic acid
FA: Fatty acid
GC: Gas chromatography
GFAP: Glial fibrillary acidic protein
GluR: Glutamate receptor
icv: intracerebroventricular
LC-MS/MS: Liquid chromatography-tandem mass spectrometry
LOQ: Limit of quantification
LTP: Long-term potentiation
NMDA: *N*-methyl-d-aspartate
OLE: Oleic acid
PC: Phosphatidylcholine
P-cPLA_2_: Phosphorylated cytosolic phospholipase A_2_
PE: Phosphatidylethanolamine
PI: Phosphatidylinositol
PrPc: Cellular prion protein
PS: Phosphatidylserine
PSD95: Postsynaptic density protein 95
PUFA: Polyunsaturated fatty acid
STD: Standard diet
