# Supplementary material for: Dietary arachidonic acid increases deleterious effects of amyloid-β oligomers on learning abilities and expression of AMPA receptors: putative role of the ACSL4-cPLA2 balance
Source: Alzheimers Res Ther. 2017 Aug 29;9:69. doi: 10.1186/s13195-017-0295-1 (PMC5576249; doi:10.1186/s13195-017-0295-1)
Supplement: Supplementary file 1 — Composition of the Aβ42 oligomer preparations. The Aβ42 oligomer preparation was separated by electrophoresis on nondenaturating 10% polyacrylamide gels. Monomers (4.6 kDa), dimers (9.2 kDa), trimers (13.8 kDa), and tetramers (18.5 kDa) were observed after Coomassie Blue staining. (PPTX 308 kb) [file 13195_2017_295_MOESM1_ESM.pptx]

## Slide 1
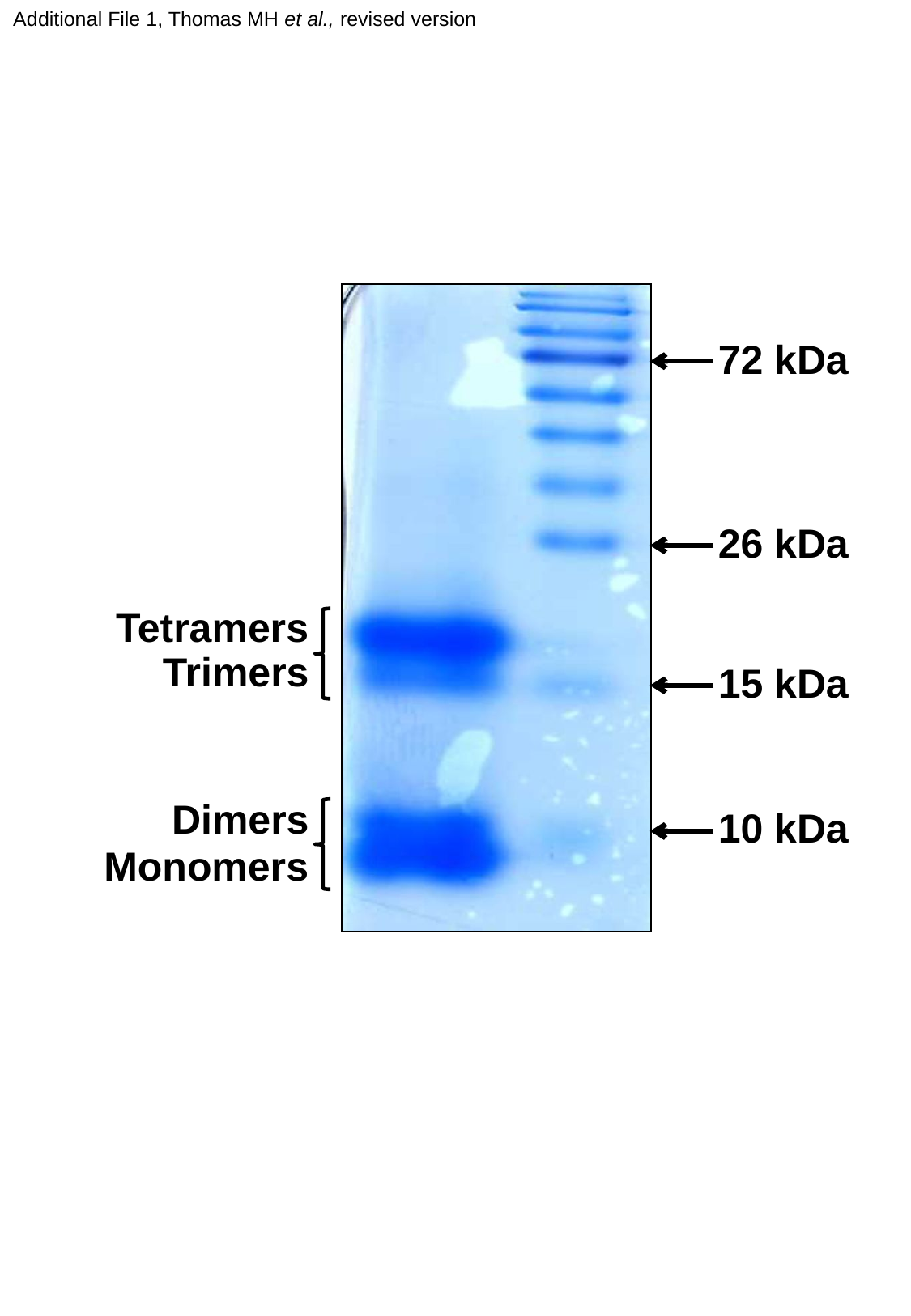

Additional File 1, Thomas MH et al., revised version
72 kDa
26 kDa
15 kDa
10 kDa
Tetramers
Trimers
Dimers
Monomers
