# Supplementary material for: Dietary arachidonic acid increases deleterious effects of amyloid-β oligomers on learning abilities and expression of AMPA receptors: putative role of the ACSL4-cPLA2 balance
Source: Alzheimers Res Ther. 2017 Aug 29;9:69. doi: 10.1186/s13195-017-0295-1 (PMC5576249; doi:10.1186/s13195-017-0295-1)
Supplement: Supplementary file 2 — Cholesterolemia levels after oleic acid-enriched (OLE) and arachidonic acid-enriched (ARA) diets. Body weight postprandial and 6-h fasting plasma cholesterol levels were measured by ELISA after 12 weeks of ARA or OLE diets. Data are expressed in grams per liter (n = 16 per group). All data are presented as mean ± standard error of the mean (SEM). (PPTX 64 kb) [file 13195_2017_295_MOESM2_ESM.pptx]

## Slide 1
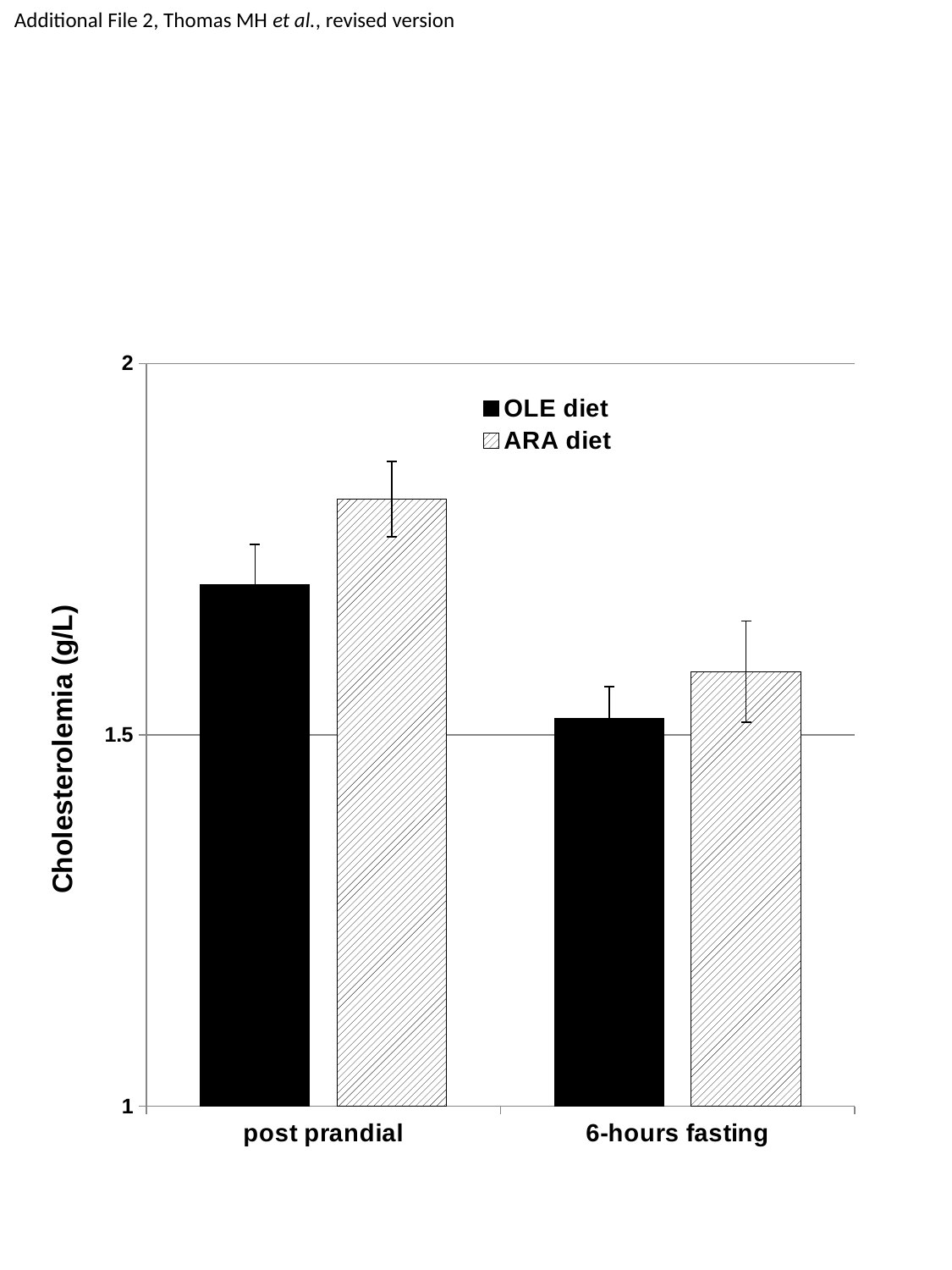

Additional File 2, Thomas MH et al., revised version
### Chart
| Category | OLE diet | ARA diet |
|---|---|---|
| post prandial | 1.7021474811129766 | 1.817400406586671 |
| 6-hours fasting | 1.5223218554983182 | 1.5853879134488482 |
