# Supplementary material for: Dietary arachidonic acid increases deleterious effects of amyloid-β oligomers on learning abilities and expression of AMPA receptors: putative role of the ACSL4-cPLA2 balance
Source: Alzheimers Res Ther. 2017 Aug 29;9:69. doi: 10.1186/s13195-017-0295-1 (PMC5576249; doi:10.1186/s13195-017-0295-1)
Supplement: Supplementary file 3 — Liver fatty acid composition in the four mouse subgroups (diets and nature of icv injections): OLE diet + NaCl injection, OLE diet + Aβ42 oligomer injection, ARA diet + NaCl injection, and ARA diet + Aβ42 oligomer injection. (DOCX 19 kb) [file 13195_2017_295_MOESM3_ESM.docx]

**Erythrocyte fatty composition in each mouse sub-groups (diets and nature of icv injections**

| **Diets** | **OLE diet** | | **ARA diet** | |
| --- | --- | --- | --- | --- |
| **icv injections** | **NaCl** | **Aβ** | **NaCl** | **Aβ** |
| **Palmitic acid 16:0** | **37.90 ± 1.47** | **37.63 ± 1.17** | **35.32 ± 1.88** | **38.07 ± 0.87** |
| **Stearic acid 18:0** | **17.28 ± 0.77** | **17.54 ± 0.52** | **19.20 ± 1.08** | **19.88 ± 0.32** |
| **Palmitoleic acid 16:1** | **0.55 ± 0.02** | **0.61 ± 0.03** | **0.41 ± 0.01** | **0.45 ± 0.02** |
| **Oleic acid 18:1** | **17.46 ± 0.29** | **17.44 ± 0.41** | **10.98 ± .022** | **11.04 ± 0.25** |
| **Linoleic acid 18:2 ω-6** | **9.03 ± 0.34** | **8.88 ± .037** | **4.61 ± 0.11** | **4.60 ± 0.25** |
| **Arachidonic acid 20:4 ω-6** | **12.20 ± 1.32** | **12.26 ± 0.62** | **23.99 ± 2.48** | **21.32 ± 0.89** |
| **Docosatetraenoic acid 22:4 ω-6** | **1.54 ± 0.18** | **1.56 ± 0.08** | **4.29 ± 0.88** | **3.39 ± 0.20** |
| **Linolenic acid 18:3 ω-3** | **0.35 ± 0.09** | **0.25 ± 0.06** | **0.29 ± 0.02** | **0.26 ± .009** |
| **Eicosapentaenoic acid 20:5 ω-3** | **0.20 ± 0.07** | **0.26 ± 0.05** | **0.00** | **0.00** |
| **Docosahexenoic acid 22:6 ω-3** | **3.48 ± 0.56** | **3.56 ± 0.36** | **1.50 ± .029** | **0.99 ± 0.08** |
| **∑ ω-6 PUFA** | **22.76** | **22.71** | **32.90** | **29.31** |
| **∑ ω-3 PUFA** | **4.03** | **4.07** | **1.79** | **1.25** |
