# Supplementary material for: Dietary arachidonic acid increases deleterious effects of amyloid-β oligomers on learning abilities and expression of AMPA receptors: putative role of the ACSL4-cPLA2 balance
Source: Alzheimers Res Ther. 2017 Aug 29;9:69. doi: 10.1186/s13195-017-0295-1 (PMC5576249; doi:10.1186/s13195-017-0295-1)
Supplement: Supplementary file 4 — Erythrocyte fatty acid composition in the four mouse subgroups (diets and nature of icv injections): OLE diet + NaCl injection, OLE diet + Aβ42 oligomer injection, ARA diet + NaCl injection, and ARA diet + Aβ42 oligomer injection. (DOCX 19 kb) [file 13195_2017_295_MOESM4_ESM.docx]

**Liver fatty composition in each mouse sub-groups (diets and nature of icv injections)**

| **Diets** | **OLE diet** | | **ARA diet** | |
| --- | --- | --- | --- | --- |
| **Injections** | **NaCl** | **Aβ** | **NaCl** | **Aβ** |
| **Palmitic acid 16:0** | **27.01 ± 1.04** | **25.87 ± 0.47** | **24.95 ± 0.32** | **24.57 ± 0.72** |
| **Stearic acid 18:0** | **14.30 ± 0.47** | **12.51 ± 0.38** | **16.29 ± 0.36** | **15.18 ± 10.61** |
| **Palmitoleic acid 16:1** | **1.13 ± 0.12** | **1.40 ± 0.10** | **0.81 ± 0.06** | **0.95 ± 0.13** |
| **Oleic acid 18:1** | **14.96 ± 1.95** | **17.43 ± 2.69** | **9.18 ± 10.47** | **10.57 ± 1.08** |
| **Linoleic acid 18:2 ω-6** | **14.49 ± 1.22** | **16.44 ± 1.33** | **12.32 ± 1.39** | **12.81 ± 31.35** |
| **Arachidonic acid 20:4 ω-6** | **16.54 ± 0.98** | **15.28 ± 0.79** | **27.46 ± 1.09** | **27.22 ± 1.39** |
| **Docosatetraenoic acid 22:4 ω-6** | **0.49 ± 0.02** | **0.44 ± 0.01** | **1.79 ± 0.24** | **2.06 ± 0.27** |
| **Linolenic acid 18:3 ω-3** | **0.36 ± 0.05** | **0.46 ± 0.05** | **0.44 ± 0.03** | **0.58 ± 0.09** |
| **Eicosapentaenoic acid 20:5 ω-3** | **0.50 ± 0.12** | **0.46 ± 0.05** | **0.07 ± 0.01** | **0.07 ± 0.03** |
| **Docosahexenoic acid 22:6 ω-3** | **10.24 ± 0.48** | **9.72 ± 0.30** | **6.70 ± 0.38** | **5.99 ± 0.45** |
| **∑ ω-6 PUFA** | **31.51** | **32.16** | **41.56** | **42.09** |
| **∑ ω-3 PUFA** | **11.10** | **10.64** | **7.20** | **6.64** |
