# Supplementary material for: Dietary arachidonic acid increases deleterious effects of amyloid-β oligomers on learning abilities and expression of AMPA receptors: putative role of the ACSL4-cPLA2 balance
Source: Alzheimers Res Ther. 2017 Aug 29;9:69. doi: 10.1186/s13195-017-0295-1 (PMC5576249; doi:10.1186/s13195-017-0295-1)
Supplement: Supplementary file 5 — Cortex and hippocampus fatty acid composition in the four mouse subgroups (diets and nature of icv injections): OLE diet + NaCl injection, OLE diet + Aβ42 oligomer injection, ARA diet + NaCl injection, and ARA diet + Aβ42 oligomer injection. (DOCX 18 kb) [file 13195_2017_295_MOESM5_ESM.docx]

**Cortex and hippocampus fatty composition in each mouse sub-groups (diets and nature of icv injections)**

| **Brain structure** | **Cortex** | | | | **Hippocampus** | | | |
| --- | --- | --- | --- | --- | --- | --- | --- | --- |
| **diet** | **OLE** | | **ARA** | | **OLE** | | **ARA** | |
| **Injection** | **NaCl** | **Aβ** | **NaCl** | **Aβ** | **NaCl** | **Aβ** | **NaCl** | **Aβ** |
| **Palmitic acid (16:0)** | **27.08 ± 0.15** | **26.75 ± 0.31** | **27.09 ± 0.36** | **27.00 ± 0.41** | **25.20 ± 0.12** | **25.26 ± 0.28** | **24.70 ± 0.41** | **24.84 ± 0.20** |
| **Stearic acid (18:0)** | **24.74 ± 0.19** | **25.11 ± 0.17** | **25.05 ± 0.21** | **25.29 ± 0.37** | **25.79 ± 0.01** | **26.27 ± 0.32** | **25.38 ± 0.63** | **26.12 ± 0.27** |
| **Palmitoleic acid (16:1)** | **0.41 ± 0.01** | **0.40 ± 0.030** | **0.39 ± 0.02** | **0.38 ± 0.04** | **0.41 ± 0.01** | **0.45 ± 0.01** | **0.41 ± 0.01** | **0.41 ± 0.01** |
| **Oleic acid (18:1)** | **12.10 ± 0.22** | **12.16 ± 0.30** | **11.51 ± 0.25** | **11.53 ± 0.21** | **12.60 ± 0.20** | **12.75 ± 0.38** | **11.39 ± 0.09** | **11.87 ± 0.23** |
| **Linoleic acid (18:2 ω-6)** | **0.62 ± 0.03** | **0.60 ± 0.02** | **0.35 ± 0.04** | **0.40 ± 0.10** | **0.61 ± 0.05** | **0.60 ± 0.01** | **0.33 ± 0.02** | **0,37 ± 0,04** |
| **Arachidonic acid (20:4 ω-6)** | **11.53 ± 0.15** | **11.47 ± 0.06** | **12.74 ± 0.18** | **12.65 ± 0.15** | **13.22 ± 0.10** | **12.94 ± 0.29** | **14.90 ± 0.40** | **14.34 ± 0.16** |
| **Docosatetraenoic acid (22:4 ω-6)** | **3.69 ± 0.04** | **3.73 ± 0.09** | **4.77 ± 0.14** | **4.72 ± 0.12** | **4.79 ± 0.30** | **4.73 ± 0.10** | **5.84 ± 0.09** | **5.79 ± 0.17** |
| **α-linolenic acid (18:3 ω-3)** | **LOQ** | **LOQ** | **LOQ** | **LOQ** | **0.11 ± 0.01** | **LOQ** | **0.03 ± 0.01** | **LOQ** |
| **Eicosapentaenoic acid (20:5 ω-3)** | **LOQ** | **LOQ** | **LOQ** | **LOQ** | **LOQ** | **LOQ** | **LOQ** | **LOQ** |
| **docosahexaenoic acid (22:6 ω-3)** | **19.83 ± 0.25** | **19.78 ± 0.22** | **18.10 ± 0.42** | **17.97 ± 0.24** | **17.28 ± 0.40** | **17.01 ± 0.39** | **16.42 ± 0.11** | **16.26 ± 0.39** |
| **Ʃ ω-6 PUFA** | **15.84** | **15.80** | **17.86** | **17.77** | **18.62** | **18.27** | **21.07** | **20.5** |
| **Ʃ ω-3 PUFA** | **19.83** | **19.78** | **18.10** | **17.97** | **17.39** | **17.01** | **16.45** | **16.26** |
| **Ratio ω-6/ω-3 PUFA** | **0.79** | **0.80** | **0.99** | **0.99** | **0.93** | **1.07** | **1.28** | **1.26** |
