# Supplementary material for: Dietary arachidonic acid increases deleterious effects of amyloid-β oligomers on learning abilities and expression of AMPA receptors: putative role of the ACSL4-cPLA2 balance
Source: Alzheimers Res Ther. 2017 Aug 29;9:69. doi: 10.1186/s13195-017-0295-1 (PMC5576249; doi:10.1186/s13195-017-0295-1)
Supplement: Supplementary file 6 — Modification of cortical AMPA receptors induced by ARA-enriched diet. Immediately after the probe test, mice were killed, and homogenates were prepared from the cortex. Representative immunoblots of cortical GluR1 (a), GluR2 (b), GluR3 (c), and GluR4 (d) from OLE or ARA mice after NaCl or Aβ42 injections are shown. Densitometric analyses were performed to determine signal intensities normalized to β-tubulin. Data are expressed as the percentage of control OLE mice injected with NaCl (* p < 0.05 comparing the four groups of mice). Results are shown as mean ± SEM of immunoblots performed for all animals (OLE groups n = 4, ARA groups n = 6). (PPTX 252 kb) [file 13195_2017_295_MOESM6_ESM.pptx]

## Slide 1
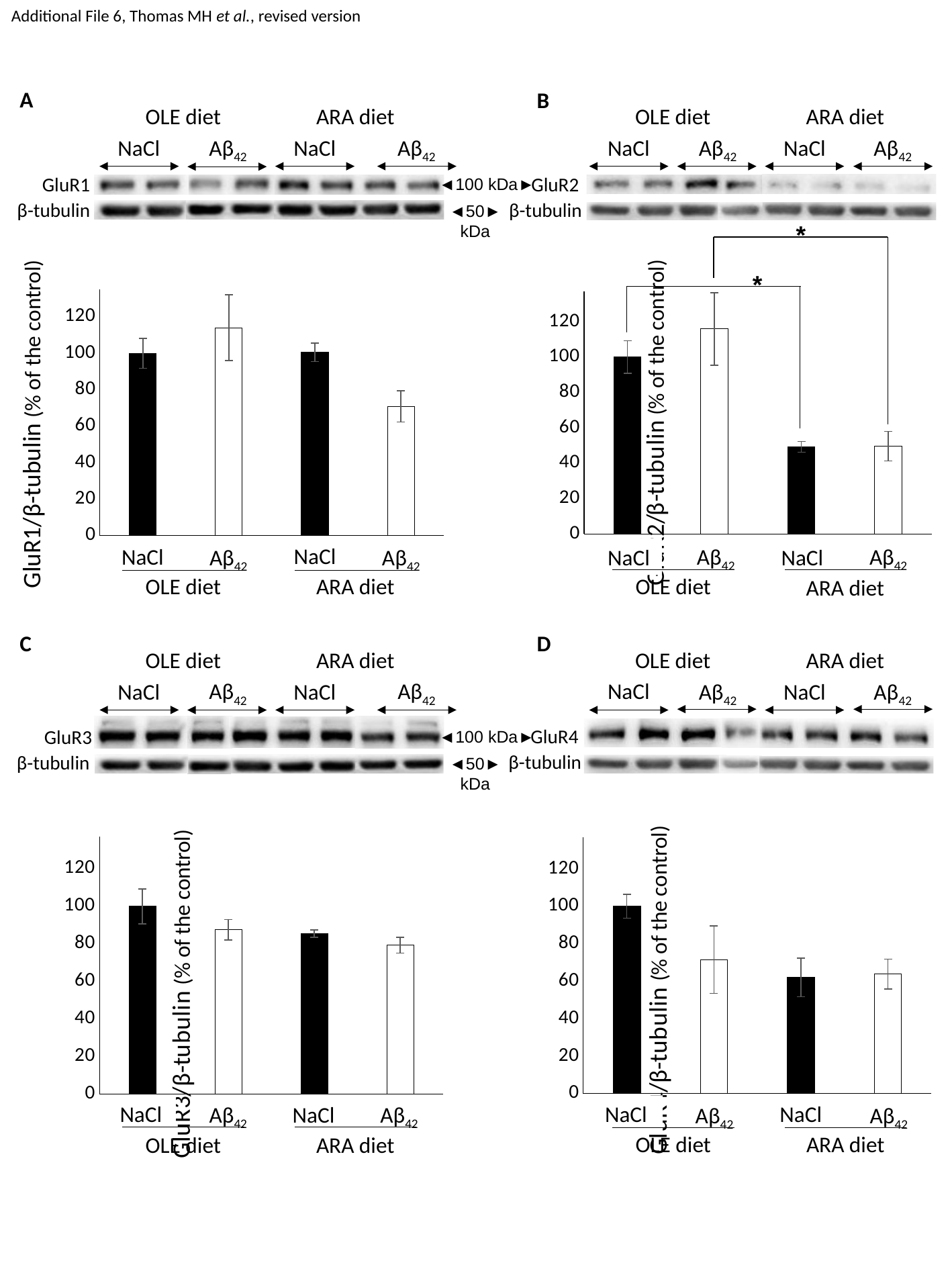

Additional File 6, Thomas MH et al., revised version
A
B
ARA diet
ARA diet
OLE diet
OLE diet
Aβ42
Aβ42
Aβ42
Aβ42
NaCl
NaCl
NaCl
NaCl
GluR1
GluR2
◄100 kDa►
 β-tubulin
 β-tubulin
◄50►
kDa
*
### Chart
| Category | |
|---|---|
| NaCl | 100.0 |
| Aβ | 114.04022279941996 |
| NaCl | 100.58085634838146 |
| Aβ | 70.87323365134789 |
### Chart
| Category | |
|---|---|
| NaCl | 100.0 |
| Aβ | 115.89856871919666 |
| NaCl | 49.27893405799979 |
| Aβ | 49.725993117489374 |*
GluR1/β-tubulin (% of the control)
NaCl
NaCl
Aβ42
Aβ42
ARA diet
OLE diet
Aβ42
Aβ42
NaCl
NaCl
OLE diet
ARA diet
C
D
ARA diet
OLE diet
ARA diet
OLE diet
NaCl
Aβ42
Aβ42
Aβ42
Aβ42
NaCl
NaCl
NaCl
GluR4
GluR3
◄100 kDa►
 β-tubulin
 β-tubulin
◄50►
kDa
### Chart
| Category | |
|---|---|
| NaCl | 100.0 |
| Aβ | 71.55546102471395 |
| NaCl | 62.0519157281404 |
| Aβ | 63.83216271330464 |
### Chart
| Category | |
|---|---|
| NaCl | 100.0 |
| Aβ | 87.47359877009622 |
| NaCl | 85.45593647596843 |
| Aβ | 79.29810704948214 |NaCl
Aβ42
Aβ42
NaCl
ARA diet
OLE diet
NaCl
NaCl
Aβ42
Aβ42
ARA diet
OLE diet
