# Supplementary material for: Dietary arachidonic acid increases deleterious effects of amyloid-β oligomers on learning abilities and expression of AMPA receptors: putative role of the ACSL4-cPLA2 balance
Source: Alzheimers Res Ther. 2017 Aug 29;9:69. doi: 10.1186/s13195-017-0295-1 (PMC5576249; doi:10.1186/s13195-017-0295-1)
Supplement: Supplementary file 9 — Modifications of brain TREM-2 proteins induced by ARA diet and Aβ42. Immediately after the probe test, mice were killed, and homogenates were prepared from the cortex and hippocampus. Representative immunoblots of cortical (a) and hippocampal (b) TREM-2 glial proteins from OLE or ARA mice after NaCl or Aβ42 injections are shown. Densitometric analyses were performed to determine signal intensities normalized to β-tubulin, and data are expressed as the percentage of control OLE mice injected with NaCl. Results are shown as mean ± SEM of immunoblots performed for all animals (OLE groups n = 4, ARA groups n = 6). (PPTX 1029 kb) [file 13195_2017_295_MOESM9_ESM.pptx]

## Slide 1
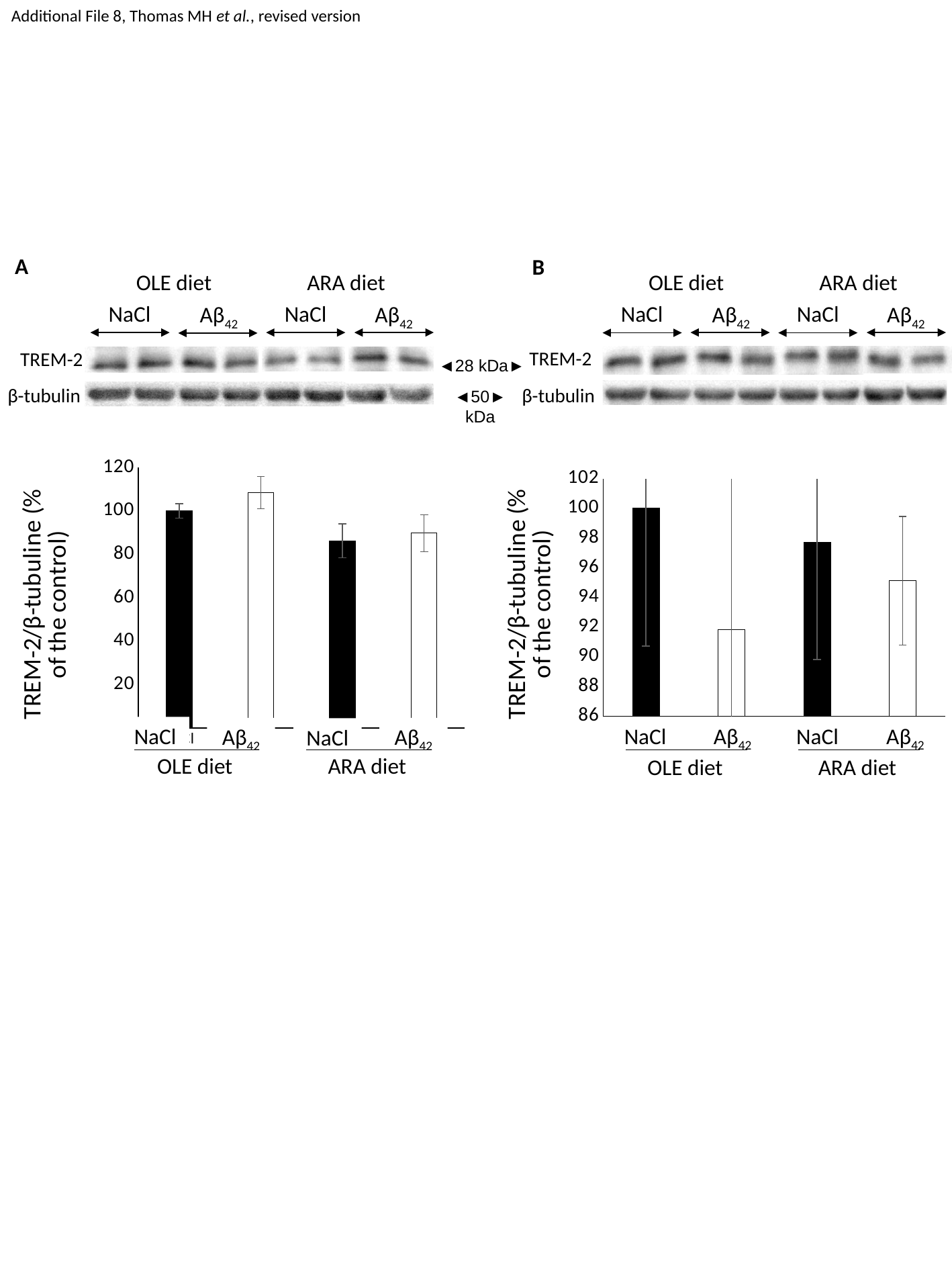

Additional File 8, Thomas MH et al., revised version
A
B
ARA diet
OLE diet
ARA diet
OLE diet
NaCl
NaCl
NaCl
NaCl
Aβ42
Aβ42
Aβ42
Aβ42
TREM-2
TREM-2
◄28 kDa►
 β-tubulin
 β-tubulin
◄50►
kDa
### Chart
| Category | |
|---|---|
| NaCl | 100.0 |
| Aβ | 108.48432111531072 |
| NaCl | 86.28953752765744 |
| Aβ | 89.77198219241764 |
### Chart
| Category | |
|---|---|
| NaCl | 100.0 |
| Aβ | 91.84127965856007 |
| NaCl | 97.70518598727953 |
| Aβ | 95.110164178761 |NaCl
Aβ42
Aβ42
NaCl
ARA diet
OLE diet
Aβ42
Aβ42
NaCl
NaCl
ARA diet
OLE diet
